# Mitochondria‐Localized Nestin Protects Mesenchymal Stem Cells from Senescence by Maintaining Cristae Structure and Function

**DOI:** 10.1002/advs.202507759

**Published:** 2025-09-24

**Authors:** Hainan Chen, Jinsi Chen, Li Huang, Xingqiang Lai, Kai Xia, Qiying Lu, Bingbing Xie, Yinong Huang, Yuan Qiu, Tao Wang, Jianqi Feng, Yuanjun Guan, Siyao Che, Jiancheng Wang, Andy Peng Xiang

**Affiliations:** ^1^ Stem Cells and Regenerative Medicine Joint Laboratory of Sun Yat‐sen University and Gaozhou People's Hospital Sun Yat‐sen University Guangzhou 510080 China; ^2^ Key Laboratory for Stem Cells and Tissue Engineering Ministry of Education Sun Yat‐Sen University Guangzhou 510080 China; ^3^ National‐Local Joint Engineering Research Center for Stem Cells and Regenerative Medicine Zhongshan School of Medicine Sun Yat‐sen University Guangzhou 510080 China; ^4^ Center of Stem Cell and Regenerative Medicine Gaozhou People's Hospital Maoming 525200 China; ^5^ Maternal‐Fetal Medicine Institute Department of Obstetrics and Gynaecology Shenzhen Baoan Women's and Children's Hospital Shenzhen 518033 China; ^6^ Department of Rehabilitation Medicine The Third Affiliated Hospital of Sun Yat‐sen University Guangzhou 510630 China; ^7^ Department of Endocrinology The First Affiliated Hospital of Sun Yat‐Sen University Guangzhou 510080 China; ^8^ Center for Stem Cells Translational Medicine Shenzhen Qianhai Shekou Free Trade Zone Hospital Shenzhen 518107 China; ^9^ Core Facilities Center for Medical Science Zhongshan School of Medicine Sun Yat‐Sen University Guangzhou 510080 China; ^10^ Department of Hepatobiliary Surgery Gaozhou People's Hospital Maoming 525200 China; ^11^ Scientific Research Center The Seventh Affiliated Hospital of Sun Yat‐sen University Shenzhen 518107 China

**Keywords:** Cellular senescence, human mesenchymal stem cells (hMSCs), Mic60, Mitochondria, Nestin

## Abstract

Nestin, a well‐characterized intermediate filament protein expressed in stem cells, is increasingly recognized for its non‐canonical roles in diverse subcellular compartments. Here, a novel mitochondrial localization of Nestin in human mesenchymal stem cells (hMSCs) is identified, where it functions as a critical protector against mitochondrial dysfunction and cellular senescence. It is demonstrated that Nestin is imported into the mitochondrial intermembrane space via its N‐terminal mitochondrial targeting sequence through Translocase of the Outer Mitochondrial Membrane 20 (TOM20)‐dependent machinery. Within mitochondria, Nestin directly interacts with Mic60 to maintain cristae architecture and sustain oxidative phosphorylation. Genetic ablation of mitochondrial Nestin triggers cristae disorganization, respiratory deficiency, and premature senescence in hMSCs. Strikingly, targeted restoration of the Mic60‐binding Tail3 domain of Nestin is sufficient to rescue cristae morphology, mitochondrial function, and senescence phenotypes. These findings establish a non‐filamentous role for Nestin in mitochondrial quality control and propose a new therapeutic strategy for age‐related disorders through modulation of mitochondrial Nestin‐Mic60 interactions.

## Graphical abstract

Nestin localizes to the mitochondrial IMS through its MTS and the TOM20 import machinery. Within the mitochondria, Nestin interacts with Mic60 via its C‐terminal Tail3 domain. Loss of mitochondrial Nestin disrupts cristae architecture, leading to mitochondrial swelling, bioenergetic dysfunction, and ultimately the senescence of hMSCs.

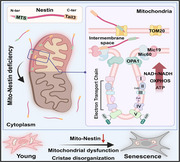

## 1 Introduction

Intermediate filaments (IFs) are dynamic structural networks within eukaryotic cells that play a critical role in regulating cell shape and behavior. They are essential for driving key cellular processes, including motility, cytokinesis, and intracellular communication.^[^ ^1^ ^]^ Within the cell, forces generated by the assembly and organization of IFs contribute to the regulation of organelles, including mitochondria.^[^ ^2^, ^3^, ^4^ ^]^ The ability of IFs to modulate cell mechanics and protect cells against compressive stress further suggests that external mechanical forces may influence mitochondrial structure and function.^[^ ^5^ ^]^ Notably, deletion of IFs proteins in cells has been shown to lead to mitochondrial fragmentation and disorganization,^[^ ^6^ ^]^ indicating that the potential for IFs proteins to play a pivotal role in regulating mitochondrial structure and metabolic function.

Nestin is a cytoskeletal protein classified as a type VI IFs.^[^ ^7^ ^]^ Initially identified in neural stem cells during brain development and in adult brains,^[^ ^8^, ^9^ ^]^ Nestin is now known to be expressed in a wide range of tissues and stem or progenitor cells, like pancreatic islets,^[^ ^10^ ^]^ skeletal muscle satellite cells,^[^ ^11^ ^]^ developing myotomes,^[^ ^12^ ^]^ testis,^[^ ^13^ ^]^ heart,^[^ ^14^ ^]^ and the bone marrow mesenchymal stromal cells (BMMSCs).^[^ ^15^, ^16^ ^]^ Like other IFs, Nestin features a central α‐helical rod domain of conserved size, flanked by a globular N‐terminus and C‐terminus domains.^[^ ^17^ ^]^ N‐terminus is required for IFs assembly, and free C‐terminus can interact with other cytoskeleton components. Due to its short N‐terminus, Nestin cannot self‐assemble into higher‐order structures and instead relies on co‐assembly, with other IFs, such as vimentin.^[^ ^17^, ^18^ ^]^ Functionally, downregulation of Nestin has been found to sensitize stem cells to oxidant‐induced cell death.^[^ ^19^ ^]^ Our group have further expanded the functional repertoire of Nestin, revealing its involvement in diverse physiological processes, such as cellular redox homeostasis, endoplasmic reticulum stress and vesicular transport.^[^ ^20^, ^21^, ^22^, ^23^ ^]^ Moreover, Nestin promotes the contact between mitochondria and endoplasmic reticulum, thereby inhibits cellular senescence.^[^ ^24^ ^]^ These findings highlight the multifaceted roles of Nestin as a key regulator of cellular biology, particularly in mitochondrial function and stem cells dynamics.

Recent studies have revealed non‐canonical functions of Nestin in subcellular compartments other than the cytosol. For instance, Nestin is observed in the nucleus and promotes cell proliferation by participating in mitotic spindle formation.^[^ ^25^, ^26^ ^]^ Nestin translocates into the nucleus through a specific signal sequence, where it helps maintain nuclear membrane integrity by binding to lamins.^[^ ^27^ ^]^ In previous work, we demonstrated that Nestin colocalizes with mitochondria using immunofluorescence, and that loss of Nestin has been shown to alter mitochondrial function, including oxygen consumption rates and ATP generation, and trigger mitochondrial morphology remodeling during stem cells differentiation.^[^ ^28^, ^29^ ^]^ Despite these advances, the relationship between the changes of Nestin's dynamic localization and its regulation of mitochondrial function during cellular processes remains poorly understood.

In this study, we reveal a previously unrecognized role of Nestin in mitochondria of human mesenchymal stem cells (hMSCs) and show that Nestin localizes to the mitochondrial intermembrane space to suppress cellular senescence. Nestin is translocated into mitochondria via the TOM20 import machinery, where it interacts with mitochondrial contact site and cristae organizing system (MICOS) to stabilize its structure. This interaction is critical for maintaining cristae morphology and supporting mitochondrial respiration in hMSCs. Our study identifies Nestin as an IF protein capable of regulating mitochondrial cristae ultrastructure through stabilizing Mic60, and provides preliminary evidence supporting further exploration of its mitochondrial interactions in aging‐related interventions.

## 2 Results

### 2.1 Nestin Localizes to the Mitochondria in hMSCs, Specifically Within the Intermembrane Space

**Figure 1:**
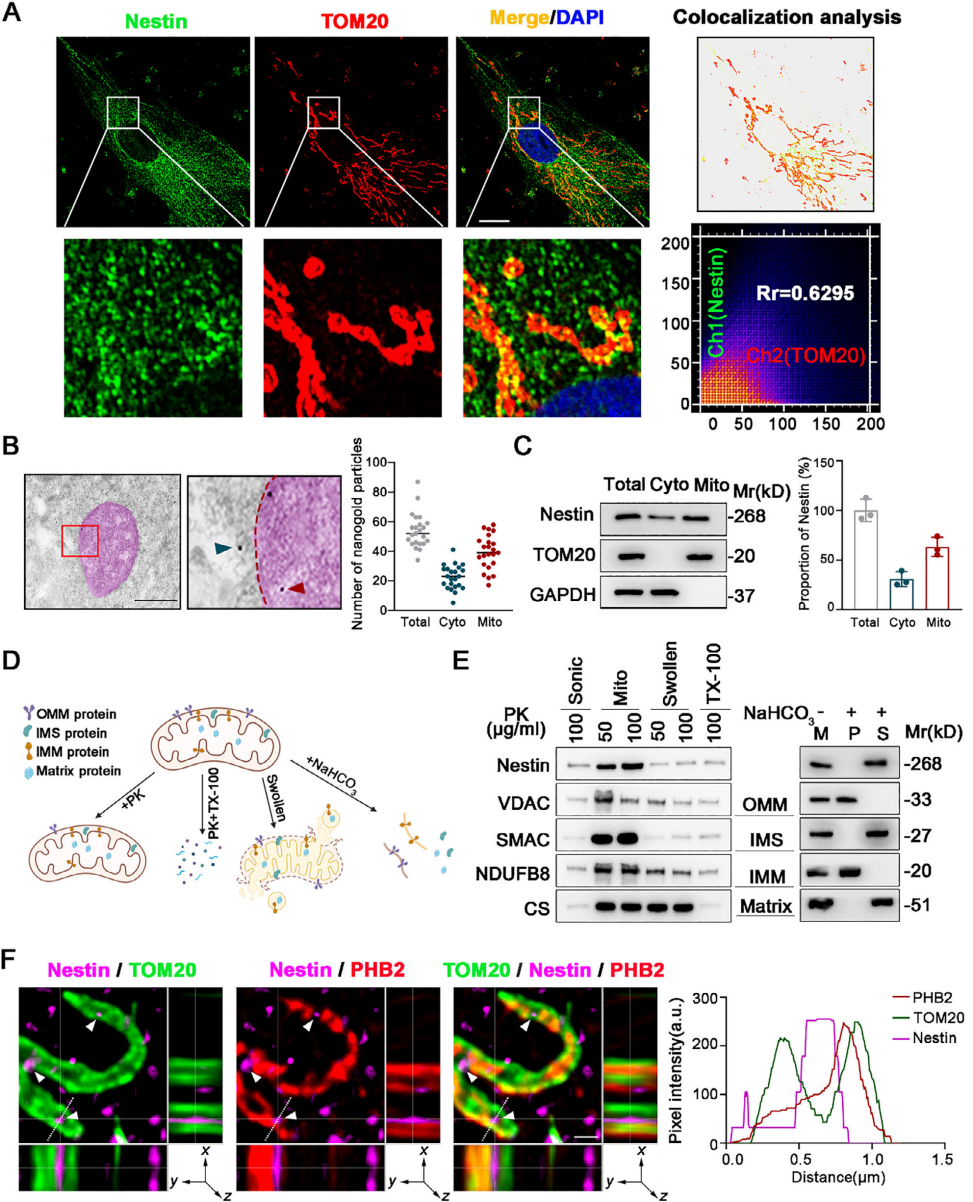
Free Nestin is localized in mitochondria of hMSCs. A) Representative images of immunofluorescence staining for Nestin and TOM20 in hMSCs. Colocalization analysis of immunofluorescence images using the colocalization plugin, which calculates Pearson’ s correlation coefficient. Scale bar,10µm. B) Representative immuno‐transmission electron microscopy (TEM) images labelled pre‐embedding with Nestin antibodies followed by nanogold‐conjugated secondary antibodies. Scale bar, 500nm. C) Western blot analysis of the expression of Nestin in subcellular fractions including total cellular (Total), cytosol (Cyto) and mitochondria (Mito) in hMSCs. Fractionation fidelity was verified by detection of GAPDH in the cytosolic fraction and TOM20 in the mitochondrial fraction. D) Schematic diagram of protease K protection assay. E) Proteinase K protection assay of Nestin in the mitochondria of hMSCs. Left: western blot analysis of Nestin after incubation of purified mitochondria with the indicated concentration of proteinase K. Right: proteinase K protection assays were performed in the presence of the permeabilizing agent NaHCO3. Extent of digestion was determined by blotting for key intra‐mitochondrial proteins (VDAC, SMAC, NDUFB8, CS). OMM, outer mitochondrial membrane; IMS, mitochondrial intermembrane space; IMM, inner mitochondrial membrane. F) Immunofluorescence Sparse‐SIM microscopy and colocalization analysis of Nestin in mitochondria. Scale bar, 0.5µm. Data are presented as the means ± SD.

Previous studies have established that Nestin is localized to multiple compartments within the cell, including the nucleus, endoplasmic reticulum, and cell membrane, among others.^[^ ^20^, ^21^, ^22^, ^23^ ^]^ This suggests that Nestin's influence on mitochondrial function may be linked to its specific subcellular positioning. To investigate this possibility, we first examined the spatial relationship between Nestin and mitochondria in hMSCs using fluorescence labeling of Nestin and Translocase of the Outer Mitochondrial Membrane 20 (TOM20). The results revealed a clear co‐localization of Nestin with mitochondria (**Figure** **1**A). To further confirm this observation, we employed immunogold electron microscopy, which demonstrated that colloidal gold particles bound to Nestin antibodies were localized inside mitochondria (Figure 1B). To complement these imaging studies, we isolated total mitochondrial and cytosolic proteins and detected the presence of Nestin in the mitochondrial fraction (Figure 1C).

To clarify the precise localization of Nestin within mitochondria, we performed protease K protection assays on mitochondrial suspensions (Figure 1D). We found that Nestin was resistant to degradation by protease K alone. However, when the mitochondrial structure was completely disrupted by the addition of Triton X‐100, Nestin was degraded. This suggests that Nestin is likely localized inside the mitochondria rather than on the outer mitochondrial membrane. Furthermore, Nestin was also degraded when protease K was applied to microparticles formed after mitochondrial swelling, indicating that Nestin is not located in the inner mitochondrial membrane or matrix. To further investigate Nestin's localization, we disrupted the mitochondrial membrane structure using an alkaline solution. After centrifugation, the sample was separated into a supernatant (S) containing proteins from the intermembrane space (IMS) and mitochondrial matrix, and a pellet (P) containing proteins from the inner and outer membranes (Figure 1E). These results suggest that Nestin is likely localized in the IMS. To confirm this finding, we performed immunofluorescence staining of Nestin, the outer mitochondrial membrane protein TOM20, and the inner mitochondrial membrane protein Prohibitin2 (PHB2). Using sparse structured illumination microscopy (Sparse‐SIM), we observed that Nestin localizes between the inner and outer mitochondrial membranes (Figure 1F). This provides further evidence that Nestin is specifically localized in the mitochondrial IMS.

### 2.2 The Translocation of Nestin to Mitochondria Depends on Its Mitochondrial Targeting Sequence and the TOM20 Import Machinery

**Figure 2:**
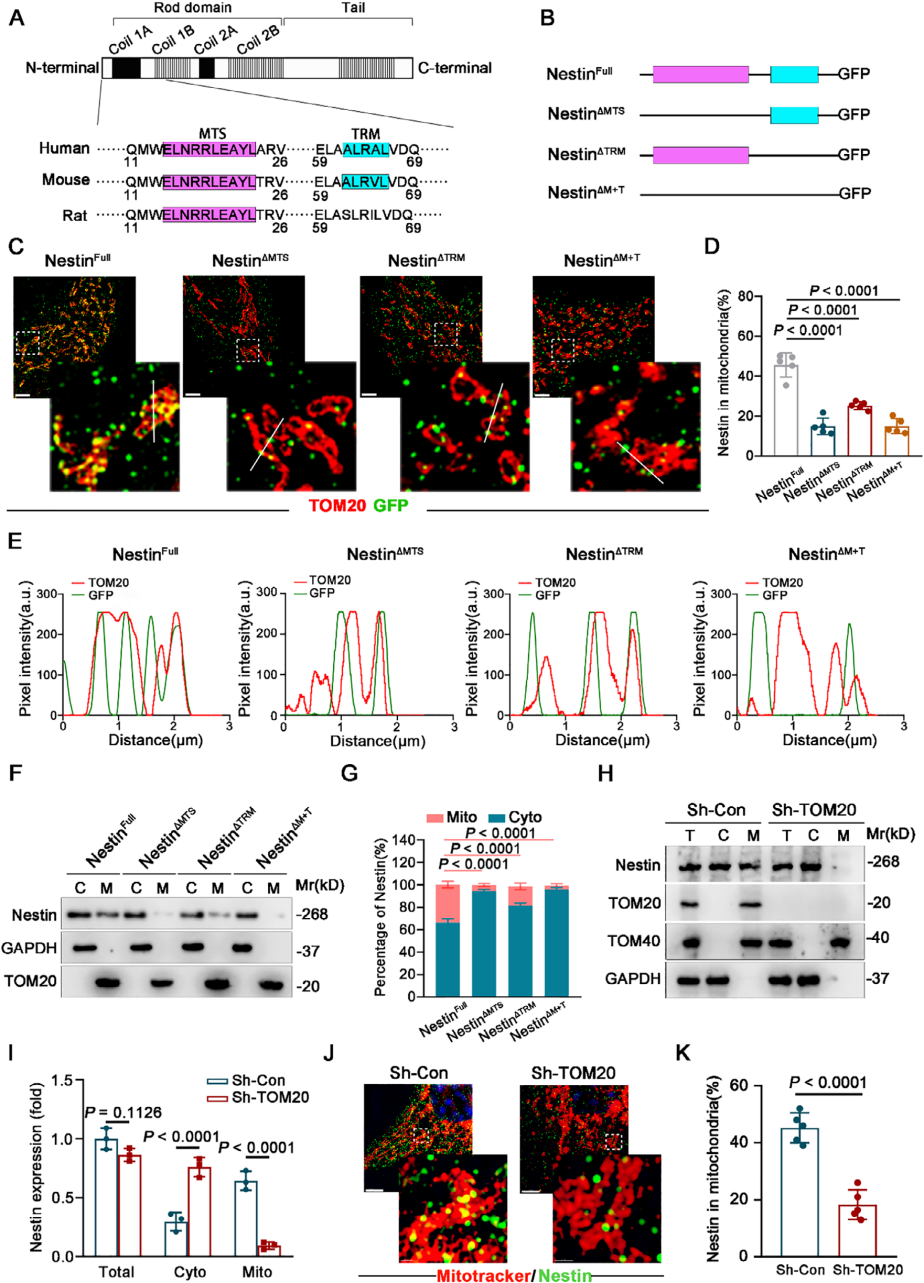
The translocation of Nestin to the mitochondria is reliant on MTS and TOM20. A) MTS and TRM identified by bioinformatic analysis. B) Schematic depiction of full length (Nestin^Full^), MTS deletion mutant (Nestin^ΔMTS^), TRM deletion mutant (Nestin^ΔTRM^), MTS and TRM deletion mutant (Nestin^ΔM+T^) of GFP‐tagged Nestin. C–E) Representative images and colocalization analysis of the Nestin (GFP) inside mitochondria (TOM20) in hMSCs overexpressed with wild‐type and deletion mutants of Nestin. Scale bar, 5µm. F,G) Western blot analysis of Nestin distribution in hMSCs (C: cytosolic lysates, M: mitochondrial lysates) and the corresponding mitochondrial/cytosolic intensity ratio of Nestin. H,I) Western blot analysis of Nestin in subcellular fractions including cytosol and mitochondria in hMSCs with knockdown of TOM20. Fractionation fidelity was verified by detection of GAPDH in the cytosolic fraction and TOM40 in the mitochondrial fraction. J,K) Immunofluorescence staining of TOM20‐knockdown hMSCs. Colocalization ratio between mitochondria and Nestin using the Manders’ coefficient as a reference index to quantify the proportion of Nestin (yellow) colocalized with mitochondria (red) relative to the total Nestin (green). Scale bar, 5µm. Data are presented as the means ± SD. Statistical differences determined with one‐way ANOVA in (D), two‐way ANOVA in (G and I) and unpaired Student's t test in (K).

To determine the molecular basis of Nestin's mitochondrial translocation, we first compared Nestin protein sequences across different species using a bioinformatics tool. We identified the presence of a Mitochondrial Targeting Sequence (MTS) and a TOM20 Recognition Motif (TRM) at the amino terminus of Nestin in humans and mice (**Figure** **2**A). To investigate the functional roles of these sequences, we constructed GFP fusion overexpression vectors with specific deletions of Nestin segments. These included the full‐length Nestin (Nestin^Full^), an MTS deletion mutant (Nestin^ΔMTS^), a TRM deletion mutant (Nestin^ΔTRM^), and a double deletion mutant lacking both MTS and TRM (Nestin^ΔM+T^) (Figure 2B). These constructs were then transfected into hMSCs. Immunofluorescence staining results demonstrated that, compared to cells overexpressing Nestin‐Full, the mitochondrial localization of Nestin was significantly reduced in hMSCs expressing Nestin^ΔMTS^, Nestin^ΔTRM^, or Nestin^ΔM+T^ (Figure 2C–E). Additionally, we isolated cytosolic and mitochondrial proteins from hMSCs to assess Nestin expression. The results confirmed that the loss of MTS and/or TRM led to a marked decrease in Nestin's translocation to mitochondria (Figure 2F,G). These findings suggest that both the MTS and TRM are critical protein sequences responsible for mediating Nestin's mitochondrial translocation.

Since TOM20 is a key protein on the outer mitochondrial membrane that facilitates the transmembrane transport of mitochondrial proteins, and Nestin contains a TRM, we investigated the role of TOM20 in Nestin's mitochondrial translocation. We assessed the expression of Nestin in the cytosol and mitochondria following TOM20 knockdown. The results demonstrated that TOM20 knockdown significantly impaired the mitochondrial translocation of Nestin (Figure 2H,I). Additionally, confocal microscopy observations revealed a marked reduction in Nestin's mitochondrial localization in TOM20‐knockdown cells (Figure 2J,K). These findings collectively indicate that the mitochondrial translocation of Nestin requires the coordinated action of the MTS and TRM on Nestin, in conjunction with TOM20.

### 2.3 The Absence of Nestin in Mitochondria Leads to Disrupted Mitochondrial Morphology and Impaired Function

To investigate the functional role of Nestin within mitochondria, we utilized the CRISPR/Cas9 gene editing system to design single‐guide RNA (sgRNA) sites targeting the fourth exon (Exon4) of the Nestin gene in human induced pluripotent stem cells (hiPSCs). Following drug selection, a monoclonal cell line with Nestin knockout (NES KO) was successfully established (Figure S1A,B, Supporting Information). Immunofluorescence staining showed that NES KO hiPSCs retained the expression of key pluripotency markers, including Nanog, SOX2, and Oct4 (Figure S1C, Supporting Information). Additionally, the proliferation capacity of hiPSCs was unaffected by the loss of Nestin (Figure S1D, Supporting Information). Teratoma formation assay identified NES KO hiPSCs demonstrated similar abilities to differentiate into the three embryonic germ layers, indicating that the knockout of Nestin does not compromise the pluripotency or differentiation potential of hiPSCs (Figure S1E, Supporting Information). Using an established hiPSCs‐neuro mesodermal progenitors (NMP)‐MSCs differentiation protocol, we generated wild‐type (WT) and NES KO hiPSCs derived MSCs (Figure S1F, Supporting Information).^[^ ^30^ ^]^ Western blot analysis confirmed Nestin ablation in NES KO hMSCs (Figure S1G, Supporting Information). Multilineage differentiation assays revealed enhanced adipogenesis but reduced osteogenesis and chondrogenesis in NES KO hMSCs compared to WT controls (Figure S1H,, Supporting Information). Flow cytometry confirmed that both WT and NES KO hMSCs expressed canonical MSCs markers (CD105, CD44, CD73, CD90) but lacked hematopoietic markers (CD34, CD45, CD19) (Figure S1I, Supporting Information). These data demonstrate that NES KO hMSCs retain MSCs identity but exhibit altered differentiation potential.

We further investigate whether the elimination of Nestin impact the morphology and functionality of mitochondria in hMSCs. Using confocal microscopy, we observed that the absence of Nestin disrupts mitochondrial structure, resulting in an a distinct morphological shift from tubular mitochondria toward enlarged, vesicular structures indicative of mitochondrial swelling (Figure S2A, Supporting Information). To further explore the functional consequences of Nestin knockout, we employed the Seahorse Cell Energy Metabolism Analyzer to assess mitochondrial energy metabolism in hMSCs. The results demonstrated that the loss of Nestin significantly reduces oxidative phosphorylation (OXPHOS) levels in these cells (Figure S2B,C, Supporting Information). Additionally, the absence of mitochondrial Nestin (mito‐Nestin) led to a marked decrease in mitochondrial ATP production and a reduced NAD^+^/NADH ratio (Figure S2D,E, Supporting Information), indicating impaired energy metabolism. Since mitochondrial energy metabolism relies on the electron transport chain (ETC), we used fluorescence quantitative PCR and Western blot to analyze the mRNA and protein expression of key ETC complex subunits, including NDUFB8 (Complex I), SDHB (Complex II), UQCRC2 (Complex III), COX2 (Complex IV), and ATP5 (Complex V). We found that Nestin knockout specifically affected the mRNA expression of mitochondrial‐encoded subunits (NDUFB8, UQCRC2, COX2, and ATP5) but did not alter the mRNA expression of the nuclear‐encoded SDHB (Figure S2F, Supporting Information). Furthermore, Nestin knockout resulted in a significant reduction in the protein levels of these ETC complex subunits (Figure S2G,H, Supporting Information), which correlated with a decrease in mitochondrial DNA (mtDNA) content (Figure S2I, Supporting Information). In addition to these findings, we observed a substantial accumulation of mitochondrial reactive oxygen species (mtROS), a decline in mitochondrial membrane potential, and an increase in mitophagy in NES KO hMSCs (Figure S3, Supporting Information). These results collectively suggest that Nestin is directly involved in regulating mitochondrial function, including maintaining structural integrity, supporting energy metabolism, and protecting against oxidative stress and mitophagy.

**Figure 3:**
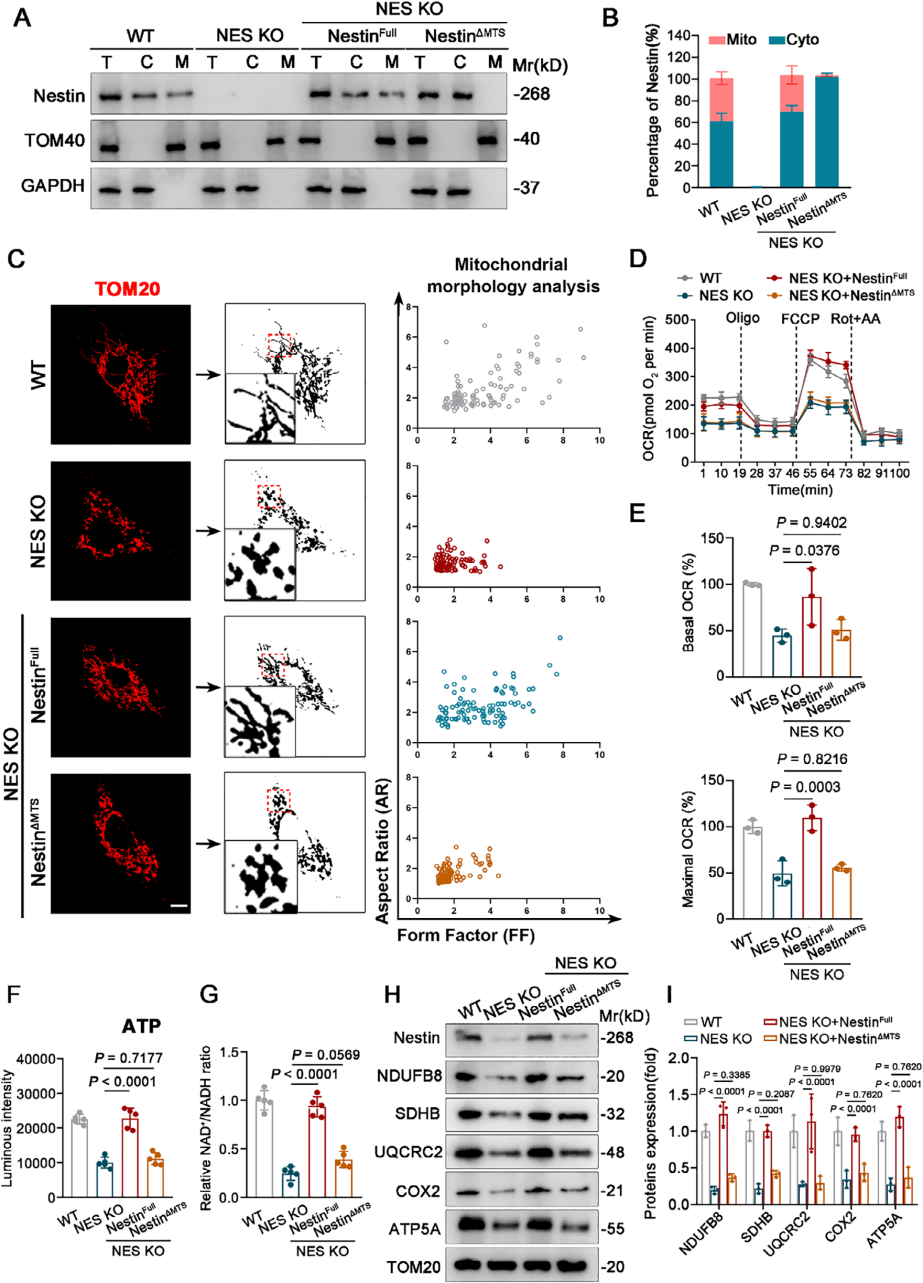
The absence of Nestin in mitochondria results in abnormal mitochondrial morphology and compromised function. A,B) Western blot analysis of the protein expression of Nestin in subcellular fractions including cytosol and mitochondria in WT hMSCs, NES KO hMSCs and NES KO hMSCs with overexpression of full length Nestin (Nestin^Full^) or Nestin mutant lacking the MTS (Nestin^ΔMTS^). C) Representative images of mitochondria in indicated hMSCs was performed using an anti‐TOM20 antibody. Scale bar, 10 µm. And mitochondrial morphology analysis by calculating the FF and AR values of representative confocal images. D,E) Seahorse XFe96 analyses of the mitochondrial respiratory capacity of indicated hMSCs (N=3 independent experiments). Oligo, Oligomycin; FCCP, carbonyl cyanide 4‐(trifluoromethoxy) phenylhydrazone; Rot+AA, Rotenon and antimycin; OCR, oxygen consumption rate. F,G) ATP production and NAD^+^/NADH ratio detected in indicated hMSCs (N=5 independent experiments). H, I) Western blot analysis of components of mitochondrial respiration complexes I(NDUFB8), II(SDHB), III(UQCRC2), IV(COX2), V(ATP5A) in indicated hMSCs. Data are presented as the means ± SD. Statistical differences determined with one‐way ANOVA in (E‐G) and two‐way ANOVA in (B and I).

To further investigate the role of Nestin in mitochondrial structure and function, we overexpressed full‐length Nestin (NES KO + Nestin^Full^) and MTS‐deficient Nestin (NES KO + Nestin^ΔMTS^) in NES KO hMSCs using lentiviral overexpression vectors. Cytosolic and mitochondrial protein fractionation demonstrated that NES KO hMSCs overexpressing full‐length Nestin exhibited mitochondrial Nestin(mito‐Nestin) localization, whereas deletion of the MTS abolished mitochondrial import – thus establishing a model of mito‐Nestin deficiency in hMSCs (**Figure** **3**A,B). Immunofluorescence staining analysis revealed that overexpression of full‐length Nestin restored mitochondrial morphology, while the mito‐Nestin deficient hMSCs failed to rescue the normal mitochondrial network. This indicates that the presence of Nestin within mitochondria is critical for maintaining mitochondrial structure (Figure 3C). Further analysis of mitochondrial dynamics revealed a marked decrease in key fusion proteins in mito‐Nestin deficient hMSCs, including reduced MFN2 expression and a lower optic atrophy protein 1(OPA1) long (L)/short (S) isoform ratio (Figure S4A, Supporting Information). For fission regulation, the phosphorylation of Drp1, a marker of mitochondrial fission, was reduced in NES KO hMSCs. No significant changes in total Drp1 or Fis1 expression was observed (Figure S4B, Supporting Information). This aligns with our prior work suggesting cytosolic Nestin influences fission,^[^ ^28^ ^]^ whereas its mitochondrial pool appears uninvolved in this process. These findings suggest that mito‐Nestin contributes to overall mitochondrial fusion by coordinately regulating the key mediators of both outer (MFN2) and inner (OPA1) membrane fusion, while appearing dispensable for fission machinery.

Using the Seahorse Cellular Energy Metabolism Analyzer, we observed that the OXPHOS levels in the mitochondria of the NES KO group and the NES KO + Nestin^ΔMTS^ group were significantly lower than those in the NES KO + Nestin^Full^ group and the control group (Figure 3D,E). Additionally, the absence of mito‐Nestin led to a substantial reduction in mitochondrial ATP production and the NAD^+^/NADH ratio (Figure 3F,G), further underscoring the importance of Nestin in mitochondrial energy metabolism. To explore the underlying mechanisms, we performed protein immunoblot to assess the expression of key ETC complex subunits. We found that the expression of these proteins was significantly lower in the NES KO + Nestin^ΔMTS^ group compared to the NES KO + Nestin^Full^ group (Figure 3H,I), suggesting that mito‐Nestin is essential for the stability and function of the ETC. These results collectively demonstrate that the translocation of Nestin into mitochondria is crucial for maintaining mitochondrial structure and function.

### 2.4 Depletion of Nestin Disrupts Cristae Homeostasis in Mitochondria

**Figure 4:**
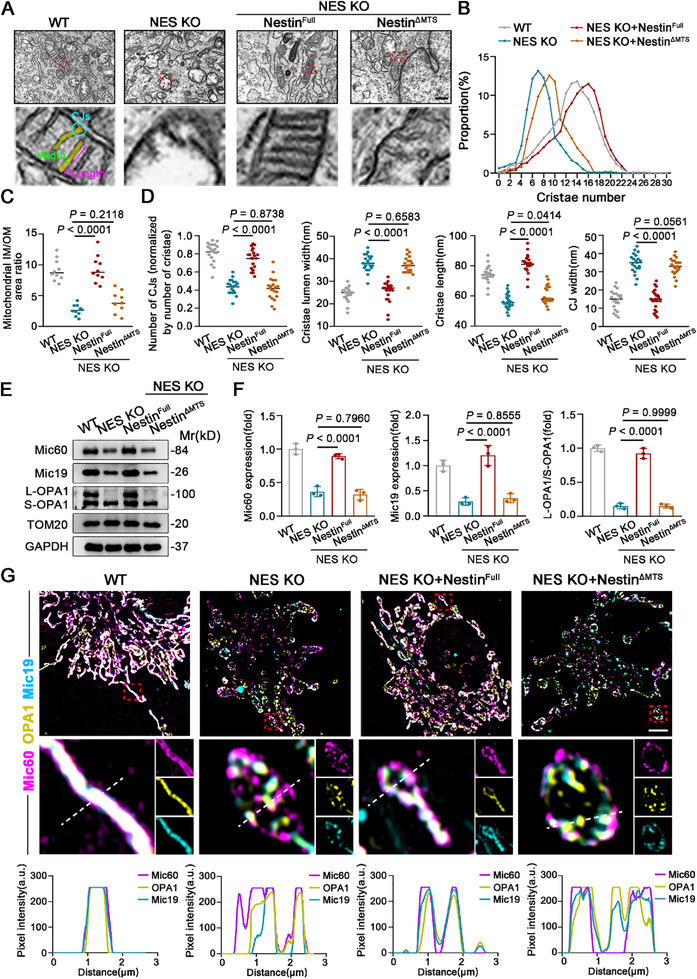
The absence of Nestin in mitochondria results in compromised Cristae homeostasis. A) Representative electron micrographs of mitochondria cristae profiles in WT hMSCs, NES KO hMSCs and NES KO hMSCs with overexpression of Nestin^Full^ or Nestin^ΔMTS^. Scale bar, 500 nm. B) Quantification of cristae number per mitochondrion and the probability distribution in indicated hMSCs. C) Quantification of the area ratio of inner membrane (IM) to outer membrane (OM) in mitochondria from indicated hMSCs. D) Quantification of the number of CJs, cristae lumen width, cristae length, and the CJs width per crista in experiments as indicated in (A). E,F) Western blot analysis of Mic60, OPA1, and Mic19 in indicated hMSCs. G) Representative images of immunofluorescence staining for Mic60, OPA1, and Mic19 by SIM in indicated hMSCs. Scale bar, 4µm. Data are presented as the means ± SD. Statistical differences determined with one‐way ANOVA.

Most of the enzymes involved in mitochondrial oxidative respiration are located on the mitochondrial cristae, which are formed by the invagination of the mitochondrial inner membrane into the matrix. The mitochondrial inner membrane plays a critical role in ATP production and maintaining the balance between NAD^+^ and NADH.^[^ ^31^ ^]^ Given Nestin's localization in the mitochondrial IMS, we hypothesized that mito‐Nestin might be involved in maintaining the stability of the mitochondrial inner membrane. Using transmission electron microscopy (**Figure** **4**A), we observed that Nestin knockout led to disorganization of the mitochondrial inner membrane structure in hMSCs, characterized by a reduction in the number of cristae and a significant decrease in the surface area of the inner membrane. These abnormalities in the mitochondrial inner membrane were effectively rescued by overexpressing full‐length Nestin but not by the MTS‐deficient Nestin mutant (Figure 4B,C). To further quantify these changes, we analyzed key morphological indicators of mitochondrial cristae, including the number of cristae junctions (CJs), cristae width, cristae length, and CJs width, based on electron microscopy data (Figure 4D). The results demonstrated that the absence of mito‐Nestin significantly disrupts the morphology and structure of the cristae. Previous studies have shown that the integrity of mitochondrial cristae is maintained by OPA1 and the mitochondrial contact site and cristae organizing system (MICOS), which is located in the IMS.^[^ ^32^, ^33^ ^]^ Protein analysis of isolated mitochondria from hMSCs revealed a decrease OPA1 L/S isoform ratio and key MICOS components, Mic60 and Mic19, following the loss of mito‐Nestin (Figure 4E,F). Additionally, confocal microscopy showed reduced interconnectivity and disorganized spatial distribution of OPA1, Mic60, and Mic19 proteins in Nestin‐deficient mitochondria (Figure 4G). Further blue native‐polyacrylamide gel electrophoresis (BN‐PAGE) analysis revealed destabilization of Mic60/Mic19‐containing complexes in mito‐Nestin deficient hMSCs versus controls (Figure S5, Supporting Information). These findings suggest that the absence of Nestin destabilizes the MICOS complex, thereby disrupting the structural equilibrium of the mitochondrial inner membrane. This highlights the critical role of Nestin in maintaining cristae integrity and overall mitochondrial function.

### 2.5 Nestin Maintains the Structural Integrity and Function of Mitochondrial Cristae Through Its Interaction with Mic60

**Figure 5:**
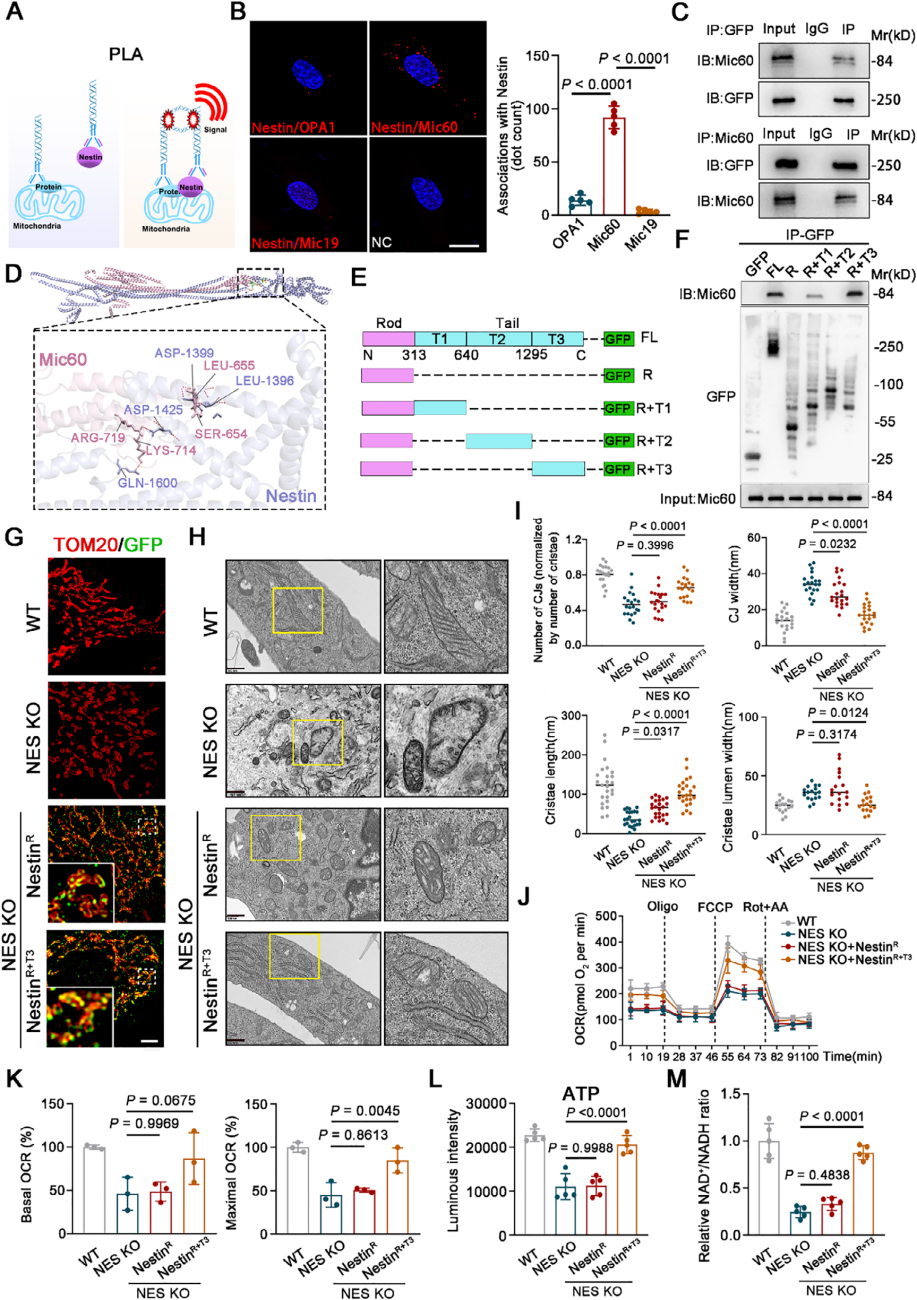
Nestin sustains the structure of mitochondrial cristae and the function of mitochondria via Mic60. A) Schematic diagram of Proximity Ligation Assay (PLA). B) Representative images and analysis were probed with a PLA (red) against Nestin with Mic60, OPA1, or Mic19 respectively in hMSCs. Scale bar, 20µm. C) Nestin‐GFP plasmids were transfected into hMSCs, whole‐cell lysates were immunoprecipitated with anti‐GFP (up) or anti‐Mic60(down), and the precipitated proteins were blotted with the indicated antibodies. D) Structural prediction of Nestin and Mic60 protein combination with AlphaFold3. Cartoon represents the predicted Nestin‐Mic60 complex structure, where the interaction hotspot residues are labeled. E) Schematic depiction of wild‐type and deletion mutants of GFP‐tagged Nestin. F) A series of truncated GFP‐tagged Nestin proteins were expressed in HEK293FT cells. Immunoprecipitation was performed using Protein G beads and an anti‐GFP antibody. G) Representative images of the Nestin fragment (GFP) inside mitochondria (TOM20) in NES KO hMSCs. Scale bar, 5µm. H) Representative electron micrographs of mitochondria in WT hMSCs, NES KO hMSCs and NES KO hMSCs with overexpression of Nestin Rod domain (R) or Nestin Rod domain linked with Tail 3 domain (R+T3). Scale bar, 500 nm. I) Quantification of the number of CJs, cristae lumen width, cristae length, and the CJs width per crista in experiments as indicated in (H). J,K) Seahorse XFe96 analyses of the mitochondrial respiratory capacity of WT hMSCs and NES KO hMSCs with overexpression of R or R+T3. (N=3 independent experiments). L,M) ATP production and NAD^+^/NADH ratio detected in indicated NES KO hMSCs (N=5 independent experiments). Data are presented as the means ± SD. Statistical differences determined with one‐way ANOVA.

To elucidate the molecular mechanism by which Nestin regulates the mitochondrial inner membrane, we investigated the interaction between Nestin and key proteins such as OPA1, Mic60, and Mic19 using Proximity Ligation Assay (PLA). PLA employs a pair of antibodies, each conjugated with oligonucleotides (**Figure** **5**A). When the target proteins are in close proximity, rolling circle amplification can be initiated, and the addition of a signal probe generates a specific fluorescence signal, indicating spatial proximity between Nestin and Mic60 (Figure 5B). Immunoprecipitation experiments further confirmed the interaction between Nestin and Mic60 (Figure 5C). Previous studies from our group have shown that Nestin is composed of multiple domains, including the Rod and Tail domains, which interact with various proteins to mediate diverse biological functions.^[^ ^20^, ^27^, ^28^ ^]^ To determine how Nestin interacts with Mic60, we resolved the structure of Nestin bound to Mic60. The results revealed that Nestin recognizes Mic60 through its Tail domain, specifically within the 1390aa‐1450aa region (Figure 5D). Given that the MTS of Nestin is located at the amino‐terminal Rod domain, we constructed overexpression vectors for protein fragments combining the Rod domain with different Tail domains (Figure 5E). Immunoprecipitation experiments demonstrated that Mic60 primarily binds to the Rod+Tail3 protein fragment of Nestin, suggesting that Nestin stabilizes the MICOS complex by interacting with Mic60 via its Tail3 domain (Figure 5F).

To further validate this, we overexpressed the Rod domain and Rod+Tail3 protein fragments in NES KO hMSCs (Figure 5G). BN‐PAGE analysis revealed that the reduced contact between Nestin and Mic60 leads to a decrease in Mic60/Mic19‐containing complexes (Figure S6, Supporting Information). Transmission electron microscopy observations revealed that overexpression of the Rod+Tail3 fragment effectively rescued the damage to the mitochondrial inner membrane caused by the absence of mito‐Nestin (Figure 5H). Additionally, analysis of mitochondrial cristae morphology showed that overexpression of the Rod+Tail3 fragment restored the disrupted cristae structure (Figure 5I). These results suggest that Nestin maintains the stability of mitochondrial cristae and mitochondrial function by binding to Mic60 through its Tail3 domain. Furthermore, analysis of energy metabolism changes demonstrated that overexpression of the Rod+Tail3 fragment significantly enhanced the level of mitochondrial OXPHOS in hMSCs compared to the NES KO group (Figure 5J,K). Concurrently, overexpression of the Rod+Tail3 fragment also increased mitochondrial ATP production and the NAD^+^/NADH ratio (Figure 5L,M), indicating that Nestin may regulate mitochondrial oxidative respiration function through its interaction with Mic60. In summary, these findings highlight that Nestin stabilizes the mitochondrial inner membrane and supports mitochondrial function by interacting with Mic60 via its Tail3 domain.

To delineate how Nestin modulates mitochondrial cristae function through Mic60, we leveraged our finding that mito‐Nestin deficiency reduces Mic60 protein levels, and mechanistically dissected Nestin's role in regulating Mic60 proteostasis. Initially, we confirmed that mito‐Nestin depletion does not significantly affect Mic60 transcription (Figure S7A, Supporting Information). Subsequent cycloheximide (CHX) chase assays to inhibit protein synthesis revealed markedly accelerated degradation of Mic60 upon disruption of its interaction with Nestin (Figure S7B,C, Supporting Information), demonstrating that mito‐Nestin protects Mic60 stabilization from proteolysis. Further investigation using the proteasome inhibitor MG132 and targeted knockdown of mitochondrial proteases (YME1L, LONP1) identified that only YME1L depletion substantially reversed Mic60 degradation in Nestin‐deficient hMSCs (Figure S7D–F, Supporting Information). Consistent with prior reports establishing Mic60 as a YME1L substrate,^[^ ^34^ ^]^ we validated through co‐immunoprecipitation that Nestin binding to Mic60 competitively inhibits YME1L‐Mic60 interaction (Figure S7G, Supporting Information). Collectively, these results establish that mito‐Nestin stabilizes Mic60 by sterically hindering YME1L‐mediated degradation (Figure S7H, Supporting Information).

To clarify the crucial role of Mic60 in how Nestin regulates mitochondrial cristae structure and function, we generated Mic60‐knockdown hMSCs (Figure S8A,B, Supporting Information) and used lentiviral vectors to overexpress either full‐length Nestin (shMic60 + Nestin^Full^) or an MTS‐deficient Nestin mutant (shMic60 + Nestin^ΔMTS^) in these cells. Consistent with published data,^[^ ^34^, ^35^, ^36^ ^]^ Mic60 knockdown disrupted mitochondrial cristae organization in hMSCs (Figure S8C, Supporting Information), manifesting as a decrease in the number of cristae, a reduction in CJs count, shorter cristae length, as well as an increase in cristae width and CJs width (Figure S8D–H, Supporting Information). Notably, overexpressing full‐length Nestin in Mic60‐deficient cells partially rescued specific parameters: it increased cristae number and reduced the width of both cristae and CJs (Figure S8D–F, Supporting Information). This partial rescue—which was not seen with the MTS‐deficient mutant—demonstrates a functional interaction between Nestin and the Mic60 pathway. However, Nestin overexpression could not restore CJs number or cristae length (Figure S8G,H, Supporting Information). In addition, analysis of energy metabolism changes showed that full‐length Nestin overexpression slightly enhanced mitochondrial OXPHOS levels compared to the shMic60 group (Figure S8I–K, Supporting Information). Concurrently, full‐length Nestin overexpression also partially increased mitochondrial ATP production and the NAD⁺/NADH ratio (Figure S8L,M, Supporting Information). These results suggest that mito‐Nestin regulates mitochondrial cristae structure and function primarily through Mic60, but may also involve additional cristae‐associated proteins.

### 2.6 Loss of Nestin in Mitochondria Accelerates the Senescence of hMSCs

Mitochondrial dysfunction has been closely linked to the senescence of human stem cells.^[^ ^37^, ^38^ ^]^ We observed that NES KO hMSCs exhibited reduced proliferative capacity compared to WT hMSCs, as evidenced by early‐onset growth arrest, a decreased percentage of S‐phase cells, impaired clonal expansion ability, and a reduction in Ki67‐positive cells (Figure S9A–D, Supporting Information). Additionally, NES KO hMSCs exhibited accelerated senescence, as evidenced by an increased proportion of SA‐β‐gal‐positive cells, upregulation of senescence‐associated secretory phenotype (SASP) factors (IL‐1β, IL‐6, IL‐8, MCP‐1), and elevated expression of senescence markers, including CDKN2A (p16) and DNA damage markers, such as γH2AX (Figure S9E–H, Supporting Information). To explore the relationship between mito‐Nestin and hMSCs senescence, we assessed Nestin levels in hMSCs using Western blot and immunofluorescence staining. We found that Nestin expression was significantly reduced in senescent hMSCs (Figure S10, Supporting Information). To evaluate the physiological relevance of mito‐Nestin in senescence beyond cellular models, we utilized PDGFRα‐creER;Rosa26‐CAG‐tdTomato reporter mice. Immunofluorescence staining of bone marrow sections showed decreased co‐localization of mito‐Nestin in PDGFRα⁺ BMMSCs from aged (24‐month‐old) mice compared to young (2‐month‐old) controls. This reduction was particularly evident in PDGFRα⁺ cells that were positive for the DNA damage marker γH2AX (Figure S11A,B, Supporting Information). Additional validation using isolated PDGFRα⁺ cells further confirmed a significant reduction in mito‐Nestin signal in senescent BMMSCs, which is consistent with our in vivo observations (Figure S11C,D, Supporting Information). Collectively, these results suggest a strong correlation between mito‐Nestin expression and cellular senescence.

**Figure 6:**
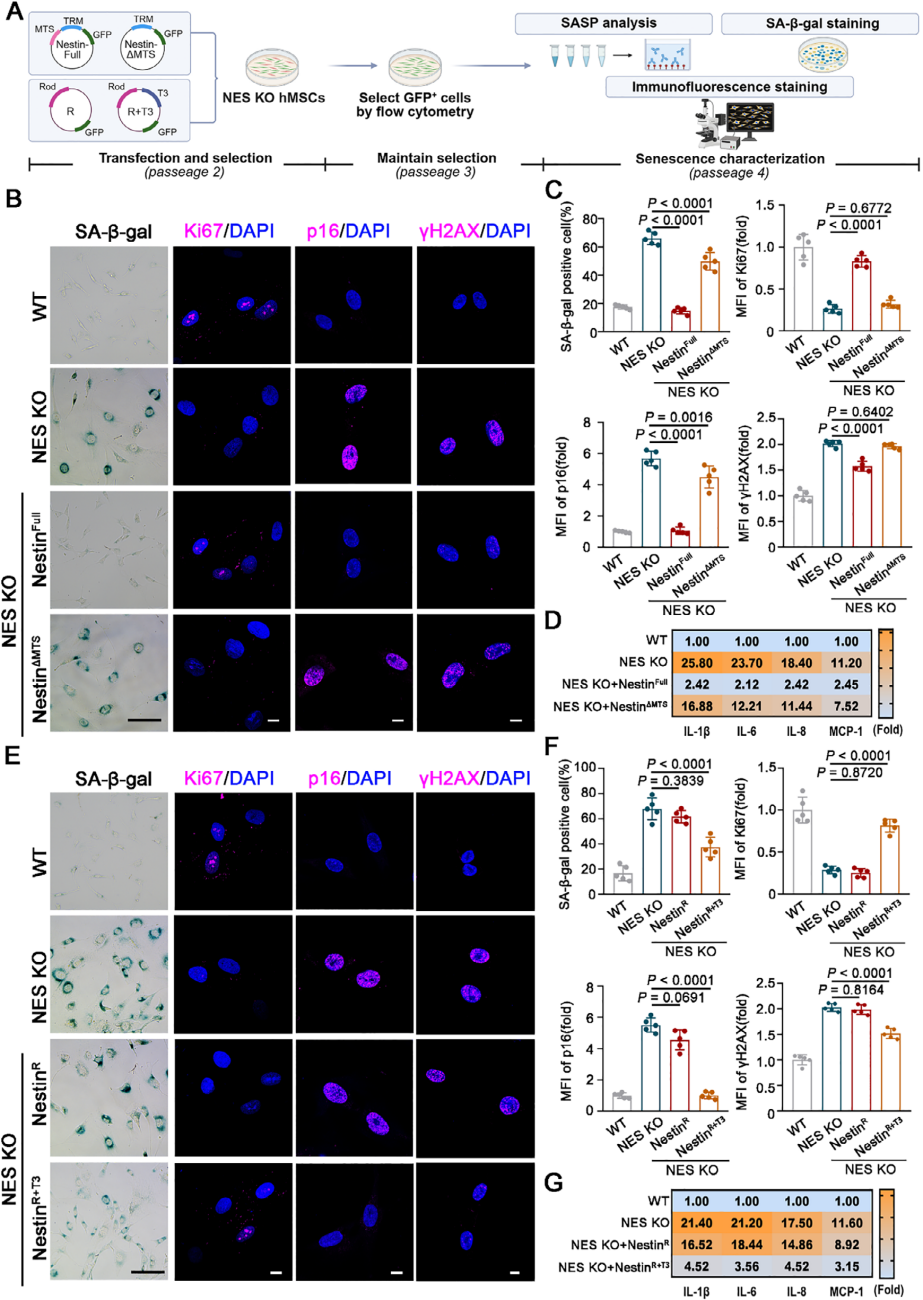
The absence of Nestin in mitochondria accelerates the senescence of hMSCs. A) Schematic diagram of NES KO hMSC infection and senescence characterization. B,C) Representative images and quantification of SA‐β‐gal staining, Ki‐67, p16 and γH2AX expression in WT hMSCs, NES KO hMSCs and NES KO hMSCs with overexpression of Nestin^Full^ or Nestin^ΔMTS^. Scale bar, 50µm (SA‐β‐gal staining);10µm (immunostaining). D) ELISA showing IL‐1β, IL‐6, IL‐8, and MCP1 secretion in indicated hMSCs. Data were shown by fold change and normalized to the WT hMSCs group. E, F) Representative images and quantification of SA‐β‐gal staining, Ki‐67, p16 and γH2AX expression in WT hMSCs, NES KO hMSCs and NES KO hMSCs with overexpression of Nestin Rod domain (Nestin^R^) or Nestin Rod domain linked with Tail 3 domain (Nestin^R+T3^). Scale bar, 50µm (SA‐β‐gal staining);10µm(immunostaining). G) ELISA showing IL‐1β, IL‐6, IL‐8, and MCP1 secretion in indicated hMSCs. Data were shown by fold change and normalized to the WT hMSCs group. Data are presented as the means ± SD. Statistical differences determined with one‐way ANOVA in (C and F).

Further, we overexpressed full‐length Nestin (NES KO + Nestin^Full^) and MTS‐deficient Nestin (NES KO + Nestin^ΔMTS^) or the Rod domain and Rod+Tail3 domain of Nestin in NES KO hMSCs using lentiviral overexpression vectors and examined cellular senescence (**Figure** **6**A). The results showed that mito‐Nestin deletion in hMSCs significantly increased SA‐β‐gal‐positive cells, reduced Ki67 expression, upregulated p16 levels, and induced DNA damage (Figure 6B,C), suggesting a strong association between mito‐Nestin and hMSCs senescence. Additionally, mito‐Nestin‐deficient hMSCs showed significantly elevated expression of SASP factors (Figure 6D). These results collectively demonstrate that mito‐Nestin plays a critical role in regulating MSCs senescence. Moreover, overexpression of the Rod+Tail3 protein fragment, but not the Rod domain alone, effectively mitigated cellular senescence in NES KO hMSCs. This was evidenced by a reduction in SA‐β‐gal‐positive cells, increased cell proliferation, less DNA damage, and suppressed expression of p16 and SASP factors (Figure 6E–G). These findings indicate that the stabilization of mitochondrial cristae homeostasis mediated by mito‐Nestin is closely linked to the regulation of MSCs senescence.

Mitochondria constitute latent triggers of cellular senescence when mtROS or mtDNA leak out of the organelle causing activation of cell cycle suppressor factors, such as p53 and p21 or cytosolic DNA sensors, such as cyclic GMP‐AMP synthase (cGAS).^[^ ^39^, ^40^ ^]^ Building upon our observations of increased mtROS accumulation and decreased mtDNA in mitochondria of NES KO hMSCs, we investigated whether mito‐Nestin regulates cellular senescence by preventing the leakage of these senescence‐inducing factors. The results confirmed significant intracellular ROS accumulation and elevated mtROS production upon mito‐Nestin deficiency. This was concomitant with activation of the p53‐p21 signaling axis (Figure S12A–E, Supporting Information). Treatment with the mtROS scavenger MitoTEMPO partially rescued the senescence phenotype in mito‐Nestin absent hMSCs (Figure S12F,G, Supporting Information). Meanwhile, loss of mito‐Nestin triggered mtDNA release into the cytosol, leading to higher levels of cGAS–STING activation as measured by significantly higher levels of pTBK1 (S172), pSTING (S365), and subsequent induction of the SASP (Figure S13A–D, Supporting Information). And, inhibiting mtDNA release using the VDAC inhibitor VBIT‐4 partially attenuated hMSCs senescence (Figure S13E,F, Supporting Information). Collectively, these findings demonstrate that mito‐Nestin acts as a critical regulator of hMSCs senescence. Its loss disrupts cristae integrity, leading to leakage of mtROS and mtDNA. These factors activate multiple downstream senescence pathways, ultimately promoting cellular senescence in hMSCs.

## 3 Conclusion

**Figure 7:**
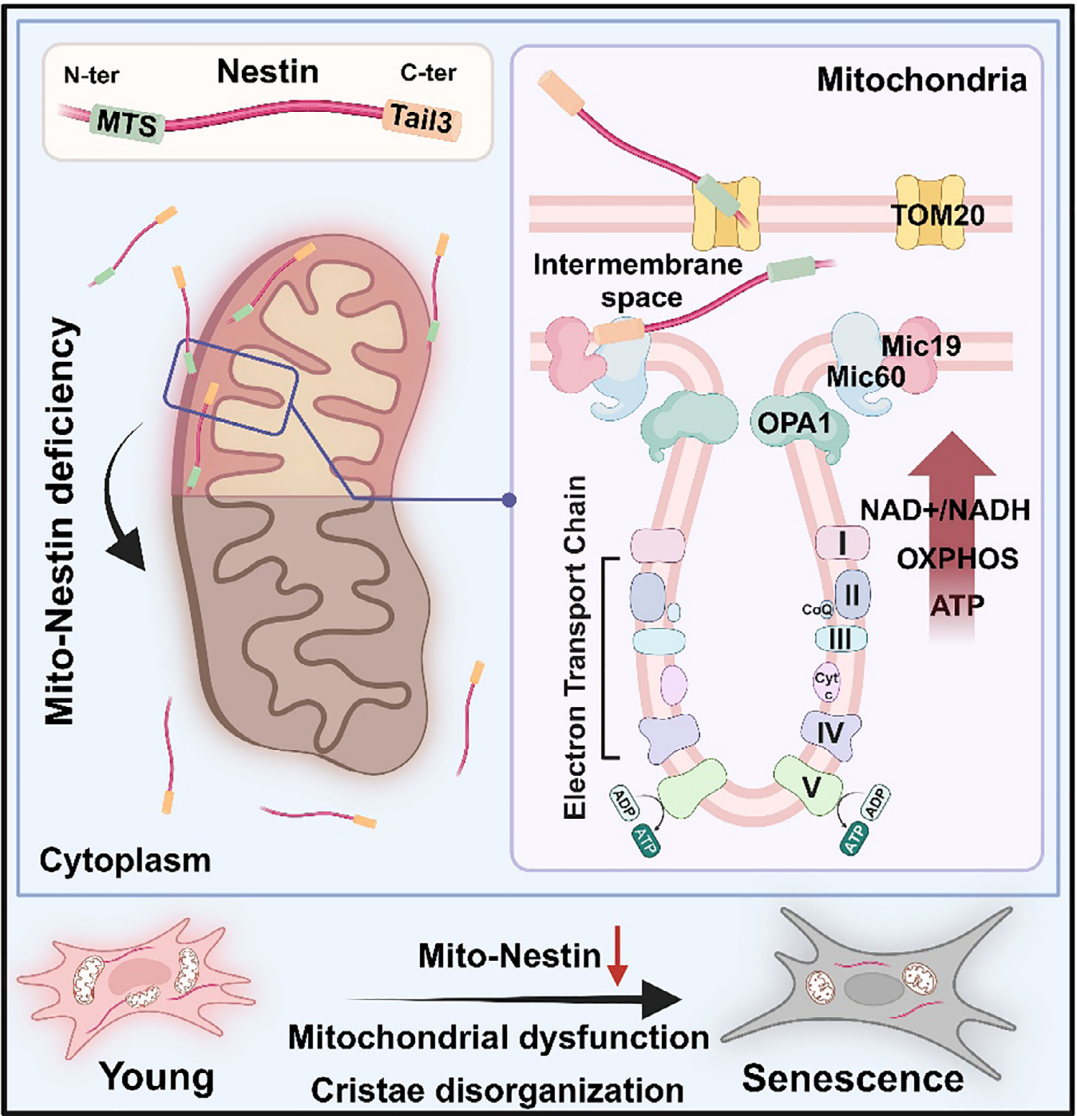
Mito‐Nestin maintains cristae structure and function: Nestin localizes to the mitochondrial IMS through its MTS and the TOM20 import machinery. Within the mitochondria, Nestin interacts with Mic60 via its C‐terminal Tail3 domain. Loss of mito‐Nestin disrupts cristae architecture, leading to mitochondrial swelling, bioenergetic dysfunction, and ultimately the senescence of hMSCs.

Our study reveals that Nestin localizes to the mitochondrial IMS through its MTS and the TOM20 import machinery. Within the mitochondria, Nestin interacts with Mic60, a key component of the MICOS, via its C‐terminal Tail3 domain. This interaction is crucial for maintaining cristae integrity, which is essential for supporting OXPHOS and overall mitochondrial function. The loss of mito‐Nestin disrupts cristae architecture, leading to mitochondrial swelling, bioenergetic dysfunction, and ultimately the senescence of hMSCs (**Figure** **7**). These findings underscore the critical role of mito‐Nestin in preserving mitochondrial structure and function, highlighting its importance in sustaining the regenerative capacity and longevity of MSCs.

IFs are known to interact with mitochondria, playing a significant role in regulating their dynamics and function.^[^ ^41^, ^42^ ^]^ For instance, the depletion of vimentin, a type of IF, disrupts mitochondrial motility, ATP production, and membrane potential, highlighting its importance in mitochondrial regulation.^[^ ^43^, ^44^ ^]^ Similarly, Nestin, another IF protein, has been shown to indirectly regulate mitochondrial dynamics through the Cdk5‐Drp1 axis, illustrating a cytoskeletal‐mediated mechanism of mitochondrial control.^[^ ^28^ ^]^ However, the direct role of cytoskeletal proteins within mitochondria remains poorly understood. In this study, we employed super‐resolution microscopy and immunoelectron microscopy to identify Nestin within mitochondria, specifically localizing to the IMS. We further demonstrated that the loss of mito‐Nestin leads to mitochondrial swelling, impaired OXPHOS, and reduced ATP production. These findings establish that mito‐Nestin plays a critical role in maintaining mitochondrial morphology and bioenergetics.

Protein import into mitochondria is a highly regulated process mediated by the translocase of the outer membrane (TOM) complex. The TOM complex consists of receptors (TOM70, TOM22, TOM20), a channel‐forming subunit (TOM40), and regulatory subunits (TOM7, TOM6, TOM5). It facilitates the recognition of cytosolic proteins, release of chaperones, and unfolding of precursor proteins for mitochondrial import.^[^ ^45^, ^46^ ^]^ Specifically, TOM20 recognizes proteins destined for the IMS by binding to their N‐terminal or internal MTS, which are characterized by hydrophobic and positively charged residues.^[^ ^47^, ^48^ ^]^ In this study, we identified both an N‐terminal MTS and a TRM in Nestin, providing a mechanistic explanation for its mitochondrial import. Deletion experiments confirmed that the MTS and TRM are essential for Nestin's interaction with the TOM complex. Furthermore, knockdown of TOM20 impaired Nestin's mitochondrial import, suggesting a TOM20‐dependent transport mechanism. The detailed molecular mechanisms underlying this process need to be further investigated.

The mitochondrial inner membrane is a critical site for ATP synthesis, maintenance of membrane potential, and selective protein transport.^[^ ^33^, ^49^, ^50^ ^]^ Damage to the inner membrane can lead to mitochondrial dysfunction and contribute to cellular pathology.^[^ ^51^ ^]^ In this study, we found that Nestin depletion reduces the surface area of the inner membrane and disrupts cristae morphology, establishing Nestin as essential for maintaining inner membrane integrity. The integrity of the mitochondrial inner membrane is regulated by key proteins such as OPA1 and the MICOS complex.^[^ ^36^, ^52^, ^53^ ^]^ The MICOS complex, particularly the Mic60‐Mic19‐Mic25 subcomplex, is essential for forming contact sites and stabilizing CJs, which are critical for maintaining mitochondrial structure and function.^[^ ^49^, ^54^, ^55^ ^]^ Conversely, the Mic10‐Mic26‐Mic27 subcomplex regulates CJs remodeling and stabilization. We observed that Nestin depletion reduces the expression of OPA1, Mic60, and Mic19 and disrupts their interactions, leading to compromised cristae morphology and structural integrity. Notably, Nestin directly interacts and protects Mic60 stabilization from proteolysis, suggesting that it may function as a mitochondrial scaffold protein. This interaction raises the possibility that Nestin plays a role in stabilizing the MICOS complex and facilitating the assembly of a larger mitochondrial intermembrane bridging (MIB) complex. The MIB complex, which may include the MICOS complex, SAM50, and other IMS proteins, is hypothesized to coordinate the alignment and structural continuity of the inner and outer mitochondrial membranes.^[^ ^56^ ^]^ Our findings suggest that Nestin may stabilize the MICOS complex and contribute to MIB assembly, thereby maintaining mitochondrial integrity. However, this hypothesis requires further investigation to fully elucidate the molecular mechanisms by which Nestin interacts with the MICOS complex and other mitochondrial proteins to regulate inner membrane structure and function. In addition, overexpression of Nestin in Mic60‐deficient cells led to a partial rescue of specific cristae parameters: it increased cristae number and reduced the width of both cristae and CJs, although it did not restore CJs number or cristae length. Both Mic60 and OPA1 are essential regulators of mitochondrial cristae architecture. Mic60 is enriched at cristae junctions and is primarily responsible for their formation and structural integrity.^[^ ^54^, ^57^ ^]^ OPA1, on the other hand, extends from the inner mitochondrial membrane into the cristae lumen, where it fine‐tunes cristae width and junction diameter.^[^ ^58^, ^59^ ^]^ Meanwhile, OPA1 is epistatic to the Mic60 in cristae shape control.^[^ ^36^ ^]^ Therefore, we propose a refined model: mito‐Nestin stabilizes cristae structure primarily by binding to and supporting Mic60 function, but also secondarily through maintaining OPA1. The loss of cristae upon Nestin depletion likely results from this combined disruption of both Mic60 and OPA1 pathways.

Cellular senescence is a key driver of organ dysfunction, impaired tissue regeneration, and chronic inflammation.^[^ ^60^, ^61^ ^]^ It arises from both intrinsic mechanisms, such as telomere attrition and genomic instability, and extrinsic stressors, including oxidative damage and immune dysfunction.^[^ ^62^, ^63^, ^64^ ^]^ Aging‐associated cytoskeletal dysfunction exacerbates cellular rigidity, oxidative stress, and telomere erosion, underscoring the cytoskeleton as a central regulator of senescence.^[^ ^65^, ^66^, ^67^ ^]^ IFs, in particular, play a critical role in cellular aging. For example, the accumulation of progeroid pre‐lamin A disrupts nuclear architecture, impairs nucleo‐cytoplasmic transport, and compromises DNA repair.^[^ ^68^, ^69^ ^]^ Similarly, lamin A/C maintains telomere positioning and stability, and its loss leads to telomere disorganization, shortening, and chromatin defects.^[^ ^65^, ^66^ ^]^ In our previous work, we demonstrated that nuclear Nestin stabilizes nuclear envelope homeostasis and delays senescence by suppressing lamin A/C phosphorylation.^[^ ^27^ ^]^ Building on this, we used a CRISPR/Cas9‐based strategy to generate Nestin‐deficient hMSCs and observed a pronounced senescent phenotype. Reintroduction of mutant Nestin further revealed that mito‐Nestin modulates hMSCs senescence by regulating mitochondrial morphology, function, and cristae integrity. These findings highlight the dual role of Nestin in cellular aging, acting both in the nucleus to maintain nuclear envelope stability and in the mitochondria to preserve cristae integrity and bioenergetic function. By linking cytoskeletal regulation to mitochondrial and nuclear homeostasis, our study provides new insights into the mechanisms underlying stem cell senescence and aging‐related tissue dysfunction.

In senescent hMSCs, mitochondrial dysfunction uniquely compromises self‐renewal and tissue regenerative capacity. This dysfunction is characterized by decreased mitochondrial membrane potential, elevated ROS production, and a reduced NAD^+^/NADH balance.^[^ ^39^, ^70^ ^]^ Specifically, senescent hMSCs exhibit downregulation of key OXPHOS subunits, including complex III components (UQCRB, UQCRFS1, MT‐CYB, UQCRC1, UQCRC2, UQCRQ), complex I subunits (NDUFA7, NDUFS4), and complex IV subunit COX7A2L.^[^ ^71^ ^]^ Disassembly of respiratory supercomplexes further impairs OXPHOS efficiency, exacerbating ROS generation and DNA damage.^[^ ^72^ ^]^ Consistent with this, we found that mito‐Nestin deficient hMSCs display reduced OXPHOS activity, accompanied by significantly diminished ATP production, NAD^+^/NADH ratio, and expression of electron transport chain (ETC) complex subunits. Otherwise, Mitochondria with compromised OXPHOS fidelity produce excessive ROS, driving oxidative damage such as base oxidation, strand breaks, and telomere attrition. These lesions activate p53/p21 and pRb pathways, inducing cell cycle arrest and senescence.^[^ ^39^ ^]^ Furthermore, dysfunctional mitochondria release damage‐associated molecular patterns, including mtDNA, which activates innate immune sensors (e.g., cGAS‐STING) and promotes inflammation.^[^ ^73^ ^]^ Here, we confirmed that mito‐Nestin deficiency triggers pronounced intracellular ROS accumulation and mtDNA leakage into the cytosol. Crucially, mito‐Nestin serves as a key regulator of hMSCs senescence: its loss disrupts cristae integrity, facilitating the release of mtROS and mtDNA. These factors converge to activate p53‐p21 and cGAS‐STING‐mediated senescence pathways, ultimately driving cellular senescence in hMSCs.

In conclusion, our study identifies Nestin as a mitochondrial protein localized to the IMS. We elucidated the molecular mechanism of Nestin's mitochondrial import, which depends on TOM20‐mediated translocation. Functionally, mito‐Nestin stabilizes Mic60 to regulate the assembly of the MICOS complex, a process essential for maintaining cristae integrity and optimizing OXPHOS efficiency. The deficiency of mito‐Nestin disrupts mitochondrial structure and function, ultimately driving hMSCs senescence. This finding underscores the importance of cytoskeletal‐mitochondrial interactions in cellular energy metabolism and highlights potential therapeutic targets for mitochondrial‐related disorders.

## 4 Experimental Section

### Cell Culture

One hiPSC line was utilized that was established in the lab as described.^[^ ^74^ ^]^ hiPSCs were cultured on Matrigel (Corning, 354234)‐coated plates in mTeSR1 medium (Stemcell Technologies) and hMSCs in ACF medium (Stemcell Technologies). The cells were passaged every 3 days using the StemPro Accutase Cell Dissociation Reagent (Life Technologies). No mycoplasma contamination was observed during cell culture. All experiments involving NES KO and WT iPSC‐MSCs—including senescence characterization—utilized early‐passage P4 cells to ensure absence of baseline replicative senescence.

### Animals

Eight‐week‐old NOG mice were purchased from Beijing Vital River Laboratory. PDGFRα‐creER mice were kindly provided by Professor Bin Zhou from Chinese Academy of Sciences. Ai9 mice (Stock NO. 007909) were purchased from the Jackson Laboratory. All animals were housed in individually ventilated cages under specific‐pathogen free (SPF) conditions in the Laboratory Animal Center of Sun Yat‐Sen University. All experimental procedures were operated in accordance with protocols approved by the Ethical Committee of Sun Yat‐Sen University.

### Generation of NES KO hiPSCs

For lenti‐CRISPR/Cas9 mediated Nestin knockout assay, sgRNA targeting Nestin (sg‐Nestin) was cloned into the lentiCRISPRv2 vector. hiPSCs were stably infected with lentiviral particles. Subsequently, NES KO hiPSCs were single cell sorted using an Influx Cell Sorter (BD) into a plastic flat‐bottomed 96 well plate, and allowed to expand. The survived clones were initially screened for Nestin gene deletion by PCR amplicons from selected genomic region on a duplicated plate and subjected to 2% agarose gel electrophoresis. Clones with negative results were further validated for Nestin KO by a western blot and were then cultured and harvested for further experiments. sgRNA sequence used for gene editing were listed in Table S3 (Supporting Information) and primers used for PCR and sequencing were listed in Table S2 (Supporting Information).

### Teratoma Formation Assay

To evaluate the tumorigenicity of NES KO hMSCs differentiated from NES KO hiPSCs, 1 × 10^6^ NES KO hMSCs in PBS containing 30% Matrigel were injected subcutaneously into 8‐week‐old NOG mice. Tumor occurrence was evaluated 8 weeks after cell injection. Animal use and all experiments involving animals were approved by the Ethical Committee of Sun Yat‐sen University (Approval NO. SYSU‐IACUC‐2018‐000034).

### Generation of NES KO hMSCs

NES KO hMSCs were differentiated from NES KO hiPSCs as previously described.^[^ ^30^ ^]^ To differentiate hiPSCs into NMP, hiPSCs were seeded on Matrigel‐coated plates and cultured in mTeSR medium for 24 h. Neuromesoderm differentiation was initiated by culturing cells in E6 medium supplemented with 20 ng mL^−1^ basic fibroblast growth factor (bFGF) and 10 µM Chir99021 (Stemgent) for 2‐5 days. For hMSCs differentiation, paraxial mesoderm cells that had been differentiated in medium with bFGF, TGFβ1, and Chir99021 for 4‐5 days were cultured for 2‐3 weeks in ACF medium. The phenotype and multipotency of NMP‐derived MSCs were assessed by FACS analysis and tested for their ability to differentiate into mesenchymal‐lineage cells (osteoblasts, adipocytes, and chondrocytes). The antibodies used for FACS were listed in Table S1 (Supporting Information).

### Isolation and Culture of Primary hMSCs

Primary hMSCs used in this study have been approved by the Institutional Review Board of Sun Yat‐Sen University (ZSYYL2017041) as previously reported.^[^ ^75^ ^]^ In brief, mononuclear cells were obtained by Ficoll‐Hypaque (1.077 g mL^−1^, Amersham Biosciences) density gradient centrifugation and seeded on Matrigel‐coated plates in ACF medium. After 3 days of culture, the medium was replaced and non‐adherent cells were discarded. At 70–80% confluence, these cells were harvested by TrypLE Express Enzyme (Thermo Scientific) for further culture.

### Plasmid Construction

For loss of Nestin function, retrovirus vectors (pSM2) encoding Nestin shRNA were used according to previous articles.^[^ ^76^ ^]^ A series of Nestin truncation mutants including Nestin^Full^(1‐1621), Nestin^R^(8‐313), Nestin^R+T1^(314‐640), Nestin^R+T2^(641‐1295), Nestin^R+T3^(1295‐1621), Nestin^△MTS^, Nsetin^△TRM^ and Nestin^△M+T^ were constructed in the laboratory. The encoding cDNAs were PCR amplified and subcloned into the pcDNA3.1‐GFP vector using appropriate restriction enzyme digests. The plasmids were listed in Table S4 (Supporting Information).

### Lentivirus Packaging

Specific plasmids were transfected into HEK293T cells together with lentiviral packing vectors including pLV/help‐SL3, pLV/help‐SL4, and pLV/help‐SL5. Then, the cell culture medium was collected at 48 h post‐transfection by ultracentrifugation at 19400 rpm at 4 °C for 2 h, and the pellets (lentiviral particles) were gently resuspended in Opti‐MEM (Thermo Scientific) medium for lentiviral transduction of hiPSCs or hMSCs.

### Mitochondria Isolation

Mitochondria were isolated with the Mitochondria Isolation Kit (Beyotime, C3601) following the manufacturer's instruction. Briefly, 2 × 10^7^ cells were lysed in cold lysis buffer from the kit for 15 min at 4 °C. The lysate was homogenized with a glass homogenizer for 30 cycles and then centrifuged at 1000 × g for 10 min at 4 °C.The supernatant was further resuspended with cell lysis reagent and centrifuged at 1000g for 10 min at 4 °C again for more purity. Last, the supernatant was collected and centrifuged at 3500 × g for 10 min at 4 °C. The pellet collected was the isolated mitochondria. Mitochondria storage reagent was used to suspend the isolated mitochondria at the required concentration for further experiments.

### Protease Protection Assay

Protease protection assay was conducted as previously described.^[^ ^77^ ^]^ Briefly, the isolated mitochondria were resuspended either in SEM buffer (250 mM sucrose,1 mM EDTA, and 10 mM MOPS pH 7.2) or, to permebalize the outer mitochondrial membrane, in hypoosmotic swelling buffer (EM buffer) (1 mM EDTA, and 10 mM MOPS pH 7.2). Proteinase K (PK) was added (final concentration 50 or 100 mg mL^−1^) and cells incubated on ice for 25 min. As controls, mitochondria were lysed by sonication or by addition of Triton X‐100 (final 1%) in the presence of PK. PK was inhibited by adding PMSF (2 mM final concentration). The samples were analyzed by western blot.

### Measurement of Mitochondrial ROS Levels and Mitochondrial Membrane Potential

The levels of mitochondrial ROS were detected using the fluorescent probes MitoSOX Red (Invitrogen). 200 µM H_2_O_2_ was used as the positive control. For measurement of mitochondrial membrane potential, cells were stained with 200 nM of tetramethylrhodamine, ethyl ester (TMRE, Invitrogen) at 37 °C for 30 min. 20 µM FCCP were used as the positive control. The cells were then washed with PBS and detected by flow cytometry. Data were analyzed by Flow Jo software.

### Seahorse Assay

Mitochondrial respiration function of cells was evaluated by measuring the oxygen consumption rate (OCR) using the XF96 extracellular flux analyzer (Seahorse Biosciences). A total of 2 × 10^4^ cells were plated on gelatin‐coated plates overnight at 37 °C. OCR was determined with the XF Cell Mito Stress Test Kit. 1 µM oligomycin, 1 µM FCCP, 0.5 µM rotenone, and 0.5 µM antimycin were used according to the recommended protocols. The basal OCR was measured before addition of respiration regulators. After injection of oligomycin and FCCP, maximal OCR was monitored. Raw values were normalized to protein concentration. For experimental design, all Seahorse XF assays were performed with N═3 biological replicates (independent cell preparations from different donors/passages), Each biological replicate included n═3 technical replicates (well replicates on the assay plate).

### Immunofluorescence Staining

For immunofluorescence staining, cells grown on coverslips were fixed with 4% paraformaldehyde (PFA) for 15 min, permeabilized with 0.1% Triton X‐100 in PBS for 20 min, and then blocked with 5% donkey serum for 30 min. After that, cells were incubated with primary antibodies overnight at 4 °C, and then a secondary antibody for 1 h at room temperature. DAPI (Roche, 10236276001) was used for nuclear DNA staining. Confocal images were captured with LSM880 and SIM imaging was performed using sparse structured illumination microscopy (Sparse‐SIM), with a lateral (XY) resolution of 60 nm and axial (Z) resolution of 200 nm. Colocalization analysis of immunofluorescence images using the colocalization plugin of ImageJ, which calculates Pearson's correlation coefficient. Colocalization ratio between mitochondria and Nestin using the Manders’ coefficient as a reference index to quantify the proportion of Nestin (yellow) colocalized with mitochondria (red) relative to the total Nestin (green). Antibodies were listed in Table S1 (Supporting Information).

### Proximity Ligation Assay (PLA)

The PLA were conducted to examine the interaction between Nestin and mitochondrial protein. Cells were fixed with 4% PFA for 10 min and blocked with 5% donkey serum for 30 min at room temperature. The PLA was performed using DUOLINK: PLA kit (Sigma‐Aldrich, Germany) as follow: the cells were incubated with specific primary antibodies and Duolink PLA Probe were added to the reaction and incubated. Subsequently, the ligation solution, consisting of two bridging DNA oligonucleotides and a ligation enzyme, was applied. Lastly, the amplification solution, consisting of nucleotides and fluorescently labeled oligonucleotides, was added together with polymerase. Control experiments included routine immunofluorescence staining of the proteins of interest under identical experimental conditions. Images were captured using a LSM880 confocal microscope (Zeiss) and analyzed by ImageJ.

### Senescence‐Associated β‐galactosidase (SA‐β‐gal) Staining

According to the manufacturer's instructions of SA‐β‐gal kit (Beyotime, C0602), cells were fixed in 4% paraformaldehyde for 10 min and incubated overnight with the working solution of βgalactosidase plus X‐Gal at 37 °C. The senescent cells were observed under an optical microscope (Leika, DMi8) and counted from five random fields of vision.

### Transmission Electron Microscopy (TEM)

Cells were fixed in 2.5% glutaraldehyde in PBS (pH 7.4) at 4 °C overnight and post‐fixed in 2% aqueous osmium tetraoxide at 4 °C for 2 h. Cells were then dehydrated in gradual ethanol (30% to 100%) and propylene oxide, embedded in EPON (Sigma‐Aldrich). Ultrathin sections of 50 nm were cut using an ultramicrotome (Leica Microsystems, UC6) with a diamond knife (Diatome, Biel, Switzerland) and stained with 1.5% uranyl acetate at 37 °C for 15 min and lead citrate solution for 4 min. For immunogold staining, cells were fixed in 4% PFA for 2‐3 h and treated with 0.5% Triton X‐100 for 40 min before sample preparation. Images were captured with FEI Tecnai G2 Spirit Transmission electron microscope.

### RNA Isolation and Real‐Time Quantitative PCR

For RNA analysis, total RNA was extracted using TRIzol Reagent (Molecular Research Center, Inc.) and processed for reverse transcription using NovoScript Plus All‐in‐one 1st Strand cDNA Synthesis SuperMix (Novoprotein). Then, real‐time quantitative PCR (qPCR) was conducted using the FastStart Essential DNA Green Master Mix (Roche, 06924204001) with the LightCycler 480 Instrument II (Roche). All samples were run in triplicate. All samples were run in triplicate and the primers used for qPCR were listed in Table S2 (Supporting Information).

### Co‐immunoprecipitation (Co‐IP) analysis

For immunoprecipitation assay, cells were lysed using Pierce IP lysis buffer (Thermo Scientific) supplemented with protease inhibitor cocktail (Roche) for 30 min on ice. The cell extracts were purified by centrifugation at 10000 × g for 10 min at 4 °C. Then the supernatants were incubated with indicated antibodies or IgG control derived from the same species as the indicated antibody overnight at 4 °C, followed by incubation with Protein G magnetic beads (Thermo Scientific) for 2 h at 4 °C. Immunocomplexes were washed twice with IP lysis buffer for subsequent immunoblot. Antibodies were listed in Table S1 (Supporting Information).

### Western Blot

In brief, cell lysates were obtained with RIPA buffer (Millipore) supplemented with protease inhibitor cocktail (Roche), and then processed for protein concentration measurement with a BCA Protein Assay Kit (Thermo Scientific). Next, equal amount of protein was prepared for SDS‐PAGE separation and electrotransferred to PVDF membranes. The target proteins were immunoblotted with specific antibodies. The primary and secondary antibodies used can be found in Table S1 (Supporting Information). Images were captured with a ChemiDoc Touch Imaging System (Bio‐Rad) and band from at least three independent blots were quantified using Image J software.

### Structural Prediction of Nestin and Mic60 Protein Combination with AlphaFold3

The structure of the Nestin‐Mic60 complex was generated from the AlphaFold3^[^ ^78^ ^]^ with the full‐length protein sequences of Nestin and Mic60 as input. Based on the template modeling and predicted alignment error values for each model, the top‐ranked structure of the protein–protein complex with the most confident score was picked out as the final model of the subsequent analysis. Prediction results were visualized using the PyMol tool.

### Blue native PAGE (BN‐PAGE)

Isolated mitochondria was permeabilized by 1.25% digitonin for 20 min and then centrifuged at 10000 g for 10 min at 4 °C. Supernatants were incorporated in BN sample buffer (Beyotime, P0761M) and loaded in 4–13% gradient blue native gel (Beyotime, P0546S) following the manufacturer's instruction. Protein complexes were bloted onto PVDF membranes and probed for the indicated antibodies or stained with Coomassie Blue (Beyotime, P0003S).

### Generation of Dox‐Inducible shRNA Clones

Tet‐On 3G Inducible Expression System, which contains pTRE3G‐IRES and pLVXTet3G vectors, was purchased from Clontech. EF1α‐Tet‐On 3G‐bsd and pTRE3G‐ shNestin‐neo were constructed using multisite Gateway technology as previously described.^[^ ^20^, ^29^ ^]^ Then, A549 cells were transfected with these two vectors, followed by selection with blasticidin and neomycin. To induce shNestin expression, the surviving A549 cells were treated with Dox.

### Statistical Analysis

All experiments were carried out with more than three biological replicates. Results were presented as means ± SEM or means ± SD. GraphPad Prism 9 software was used to conduct a two‐tailed unpaired t‐test, one‐way ANOVA or two‐way ANOVA. P values less than 0.05 were considered significant, and the level of significance was defined as *p < 0.05, **p < 0.01, and ***p < 0.001.

## Conflict of Interest

The authors declare no conflict of interest.

## Supporting information



Supporting Information

## Data Availability

The data that support the findings of this study are available from the corresponding author upon reasonable request.
