# Supplementary material for: Mitochondria‐Localized Nestin Protects Mesenchymal Stem Cells from Senescence by Maintaining Cristae Structure and Function
Source: Adv Sci (Weinh). 2025 Sep 24;12(46):e07759. doi: 10.1002/advs.202507759 (PMC12697773; doi:10.1002/advs.202507759)
Supplement: Supplementary file 1 — Supporting Information [file ADVS-12-e07759-s001.docx]

Supporting Information

Mitochondria-localized Nestin Protects Mesenchymal Stem Cells from Senescence by Maintaining Cristae Structure and Function

Hainan Chen, Jinsi Chen, Li Huang, Xingqiang Lai, Kai Xia, Qiying Lu, Bingbing Xie, Yinong Huang, Yuan Qiu, Tao Wang, Jianqi Feng, Yuanjun Guan, Siyao Che*, Jiancheng Wang*, Andy Peng Xiang*


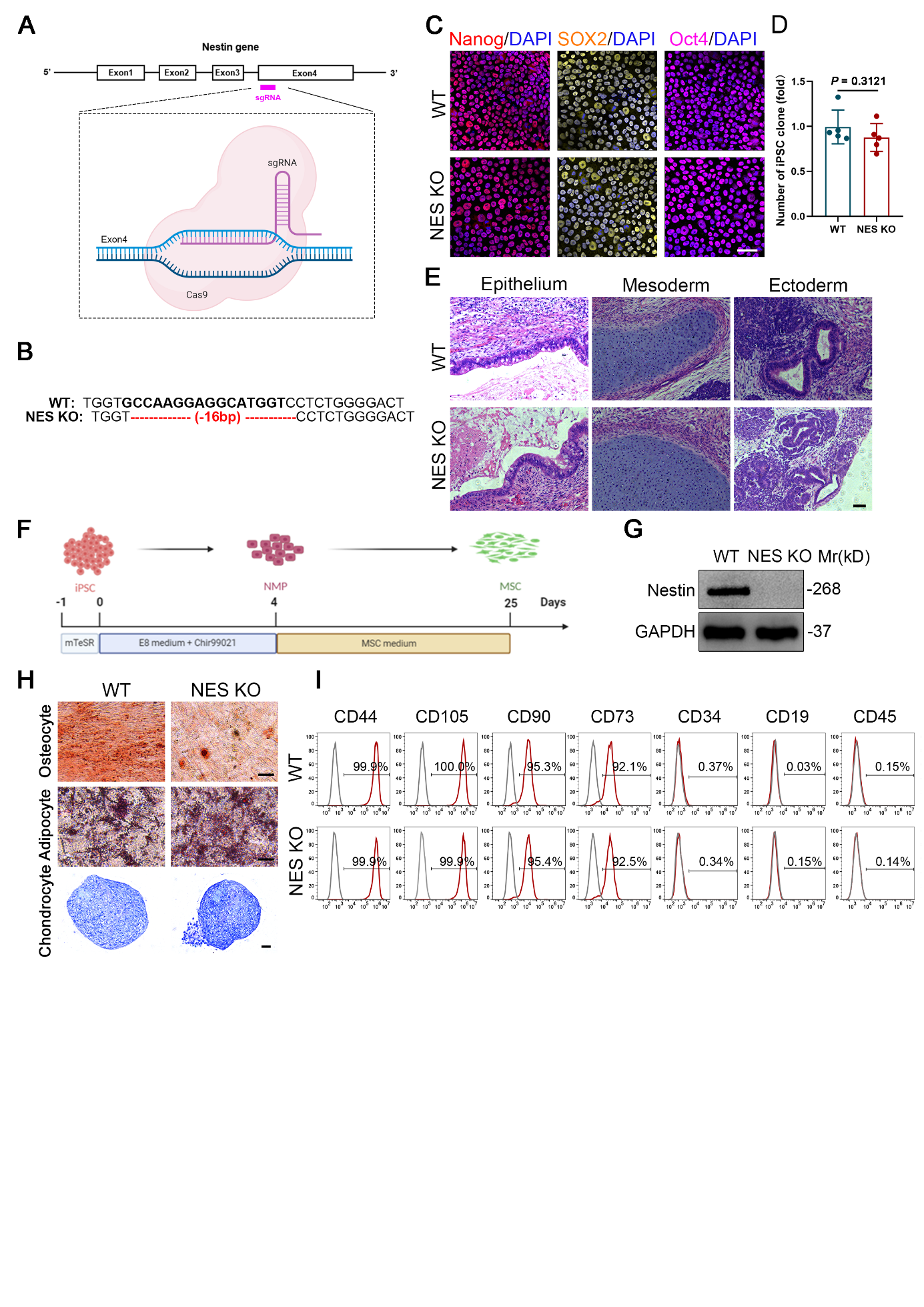


**Figure S1.** Nestin-deficient hMSCs generated from human iPSCs, related to Figure 3. A) Schematic diagram of knocking out the Nestin gene in human iPSCs (hiPSCs) using the CRISPR-CAS9 system. B) DNA sequences of the Nestin knockout site in WT and knockout hiPSCs. C) Representative confocal fluorescence images of pluripotent markers Nanog, SOX2 and Oct4 in WT and NES KO hiPSCs. Scale bars, 100 μm. D) Quantification of clone numbers of WT and NES KO hiPSCs in the similar culture state. E) Haematoxylin and eosin (H&E) staining images of the three germ layers in teratomas developed from WT and NES KO hiPSCs. Scale bar, 100μm. F) Schematic representation of the differentiation conditions used to generate MSCs from hiPSCs. G) Western blot for Nestin in WT and NES KO hMSCs. H) The osteogenic, adipogenic, and chondrogenic differentiation potentials of hMSCs were verified by Alizarin Red S staining, oil red O staining, and toluidine blue staining, respectively. Scale bars, 100 μm. I) Flow cytometry of WT and NES KO hMSCs demonstrated a typical MSCs surface pattern. Data are presented as the means ± SD. Statistical differences determined with unpaired Student’s t test.


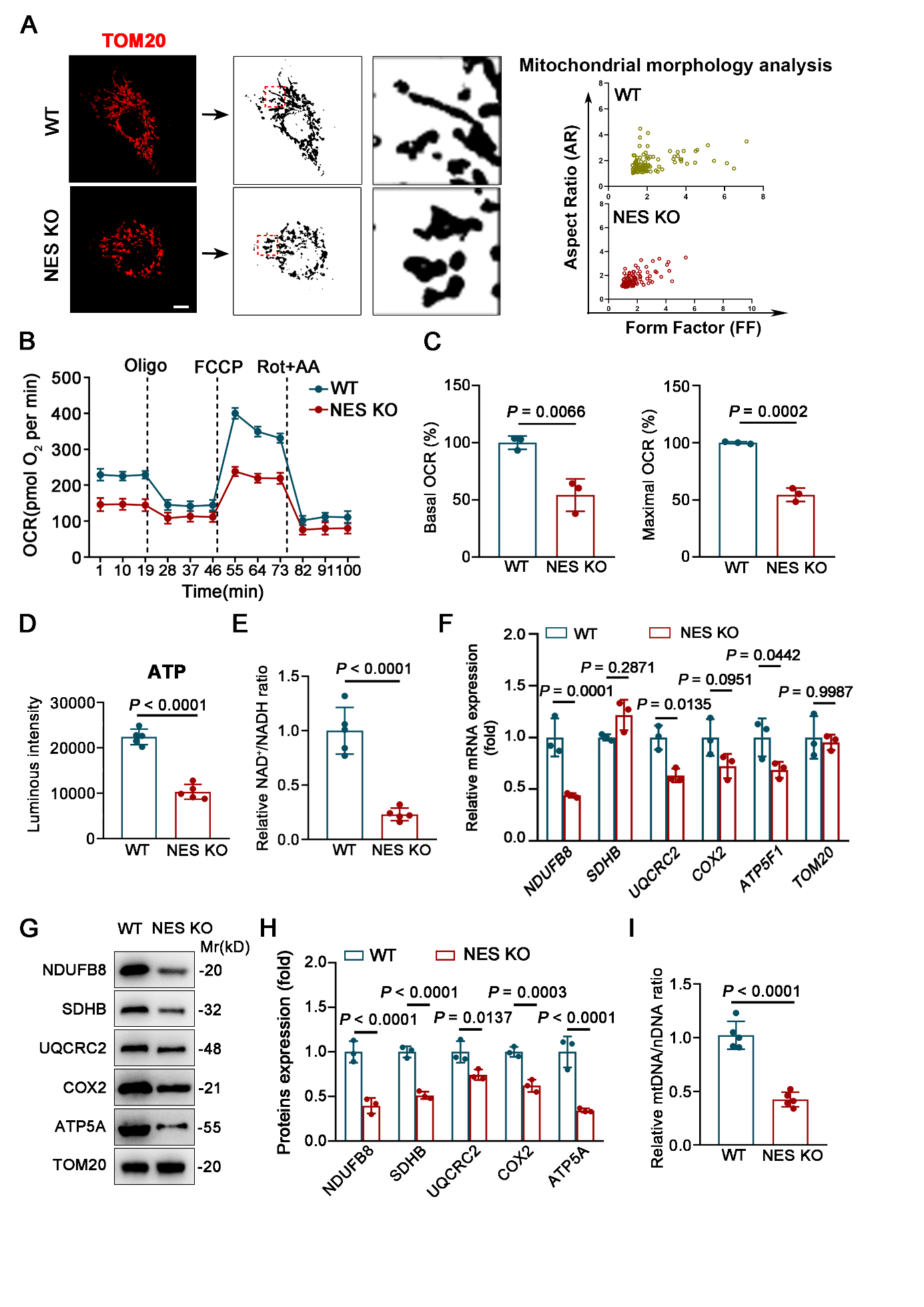


**Figure S2.** Nestin-deficient hMSCs show an abnormal mitochondrial morphology and function, related to Figure 3. A) Representative images of mitochondria in WT and NES KO hMSCs were performed using an anti-TOM20 antibody. Scale bar, 10 μm. And mitochondrial morphology analysis by calculating the FF and AR values of representative confocal images. B,C) Seahorse XFe96 analyses of the mitochondrial respiratory capacity of WT and NES KO hMSCs (N=3 independent experiments). Oligo, Oligomycin; FCCP, carbonyl cyanide 4-(trifluoromethoxy) phenylhydrazone; Rot+AA, Rotenon and antimycin; OCR, oxygen consumption rate. D, E) ATP production and NAD^+^/NADH ratio detected in indicated hMSCs (N=5 independent experiments). F) mRNA levels of components of mitochondrial respiration complexes I(NDUFB8), II(SDHB), III(UQCRC2), IV(COX2), V(ATP5A), TOM20 in WT and NES KO hMSCs analyzed by qRT-PCR. G, H) Western blot analysis of NDUFB8, SDHB, UQCRC2, COX2, ATP5A in WT and NES KO hMSCs. I) Quantitative polymerase chain reaction analysis of relative mtDNA content in WT and NES KO hMSCs. Data are presented as the means ± SD. Statistical differences determined with unpaired Student’s t test in (C-E and I) and two-way ANOVA in (F and H).


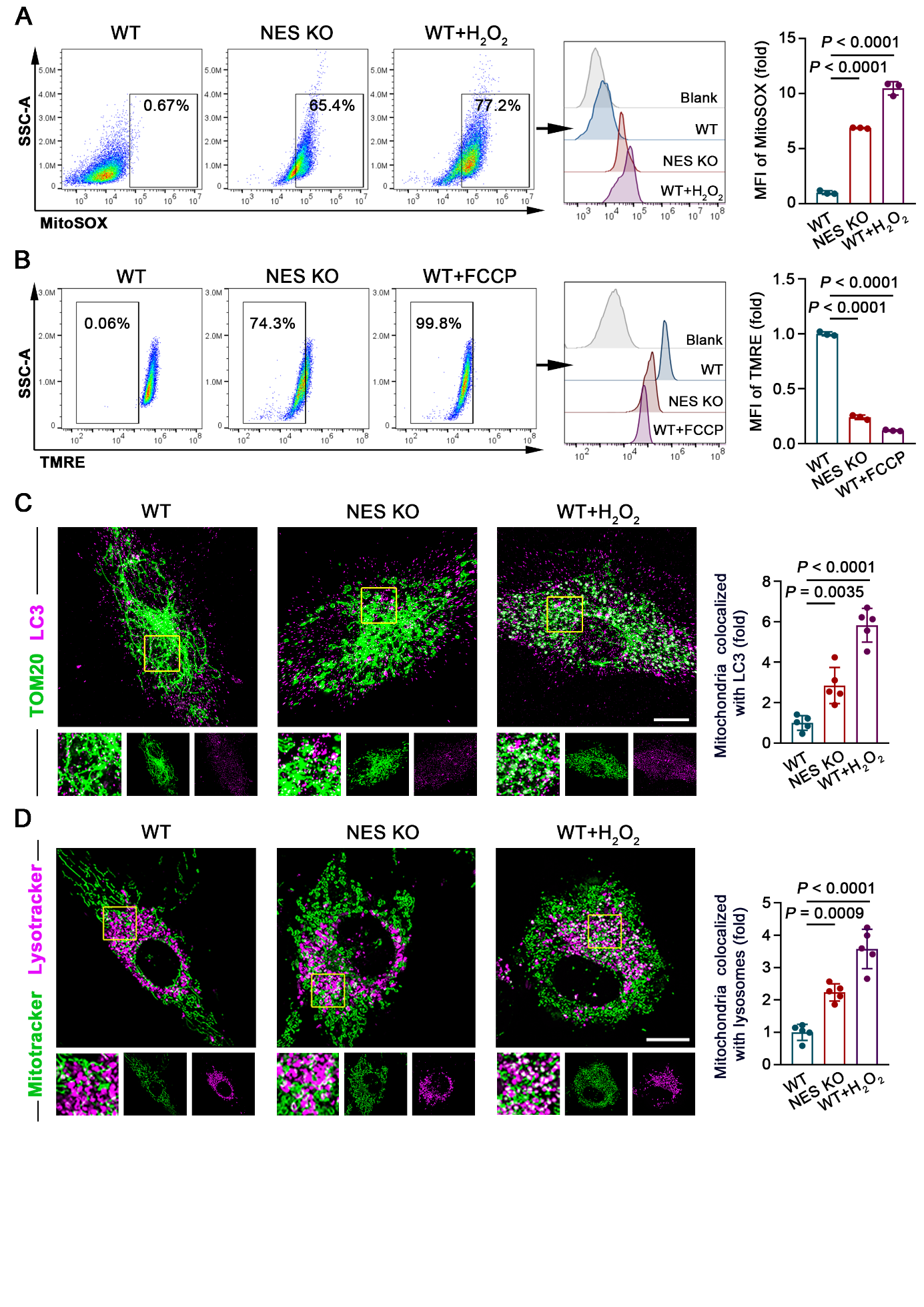


**Figure S3.** Nestin-deficient hMSCs have increased mitochondrial damage, related to Figure 3. A) Flow cytometry of mitochodnrial ROS level stained with MitoSOX of WT and NES KO hMSCs. 200 μM H_2_O_2_ was the positive control added to the WT group. B) Flow cytometry of mitochondrial membrane potential stained with TMRE of WT and NES KO hMSCs. 20 μM FCCP was the positive control added to the WT group. C) Representative confocal images of LC3 and mitochondria (TOM20) in WT and NES KO hMSCs. 200 μM H_2_O_2_ was the positive control added to the WT group. Scale bar, 10μm. D) Representative confocal images of lysosomes (Lysotracker) and mitochondria (Mitotracker) in WT and NES KO hMSCs. 200 μM H_2_O_2_ was the positive control added to the WT group. Scale bar, 10μm. Data are presented as the means ± SD. Statistical differences determined with one-way ANOVA.


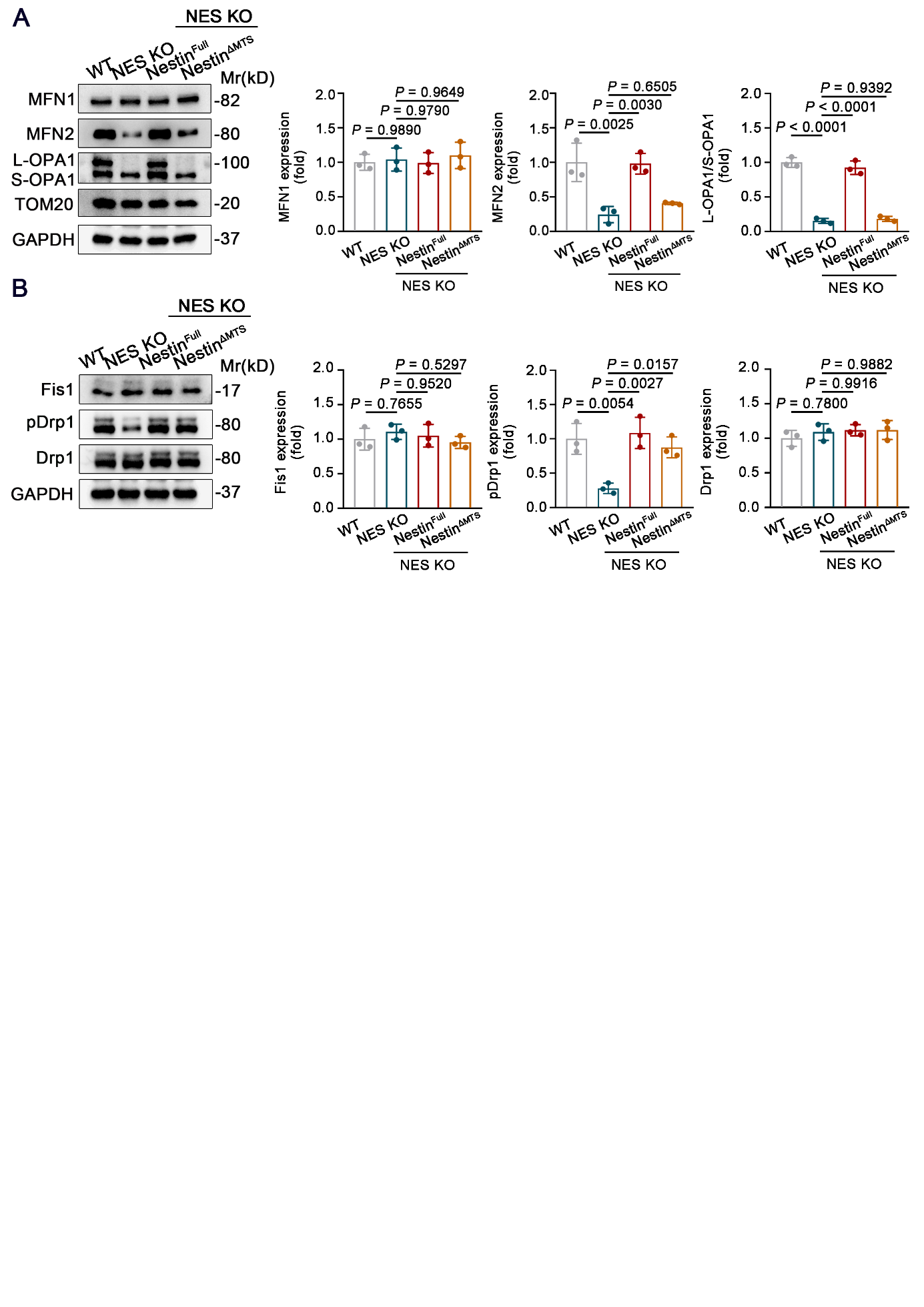


**Figure S4.** Mitochondrial Nestin deficiency affects mitochondrial dynamics, related to Figure 3. A) Western blot analysis of MFN1, MFN2 and OPA1 in indicated hMSCs. B) Western blot analysis of Fis1, pDrp1 and Drp1 in indicated hMSCs. Data are presented as the means ± SD. Statistical differences determined with one-way ANOVA.


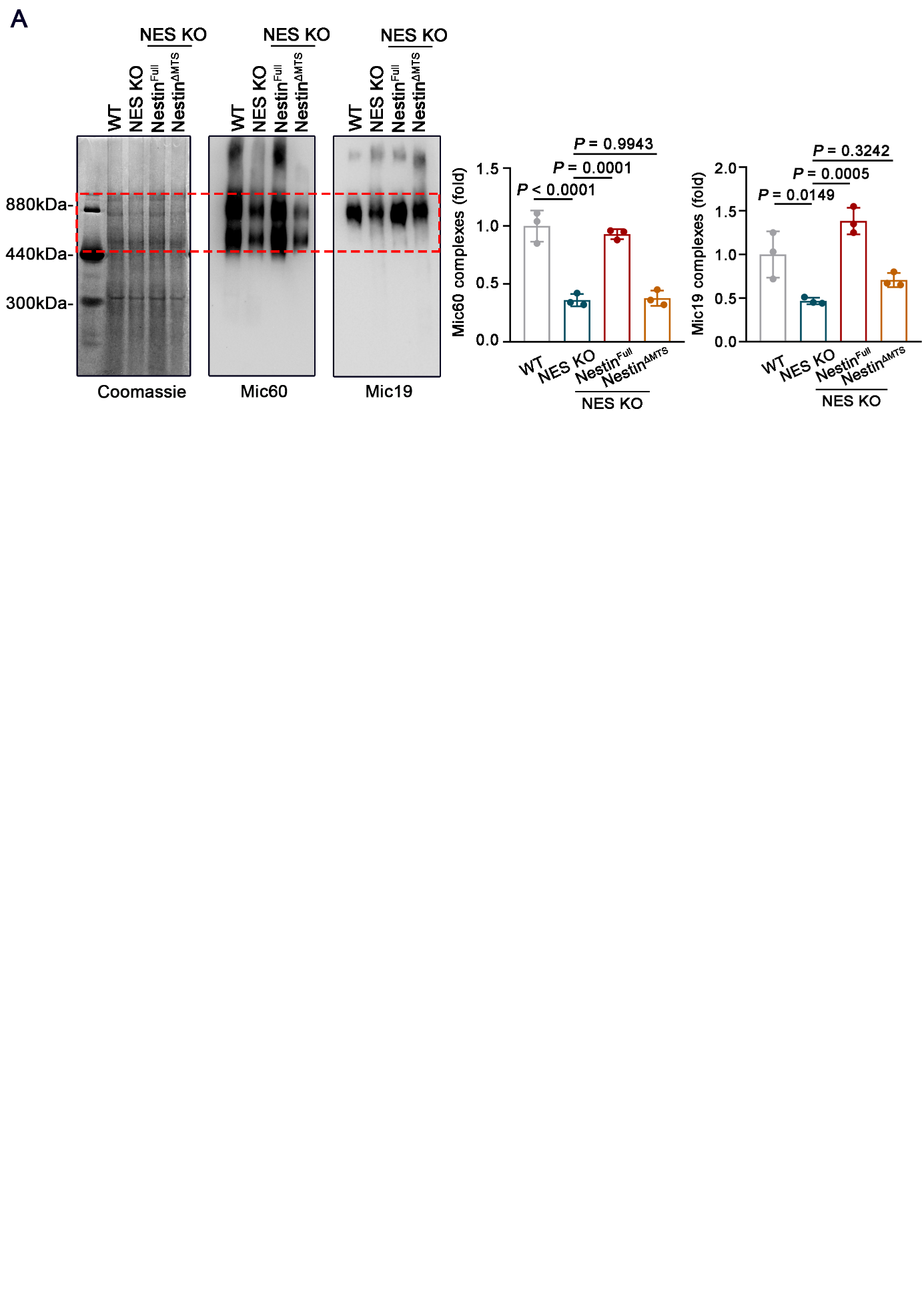


**Figure S5.** Mitochondrial Nestin deficiency destabilizes the MICOS complex, related to Figure 4. A) Protein complexes extracted from mitochondria treated as indicated were separated by BN-PAGE, Coomassie stained or transferred onto PVDF membranes, and probed using the indicated antibodies. Data are presented as the means ± SD. Statistical differences determined with one-way ANOVA.


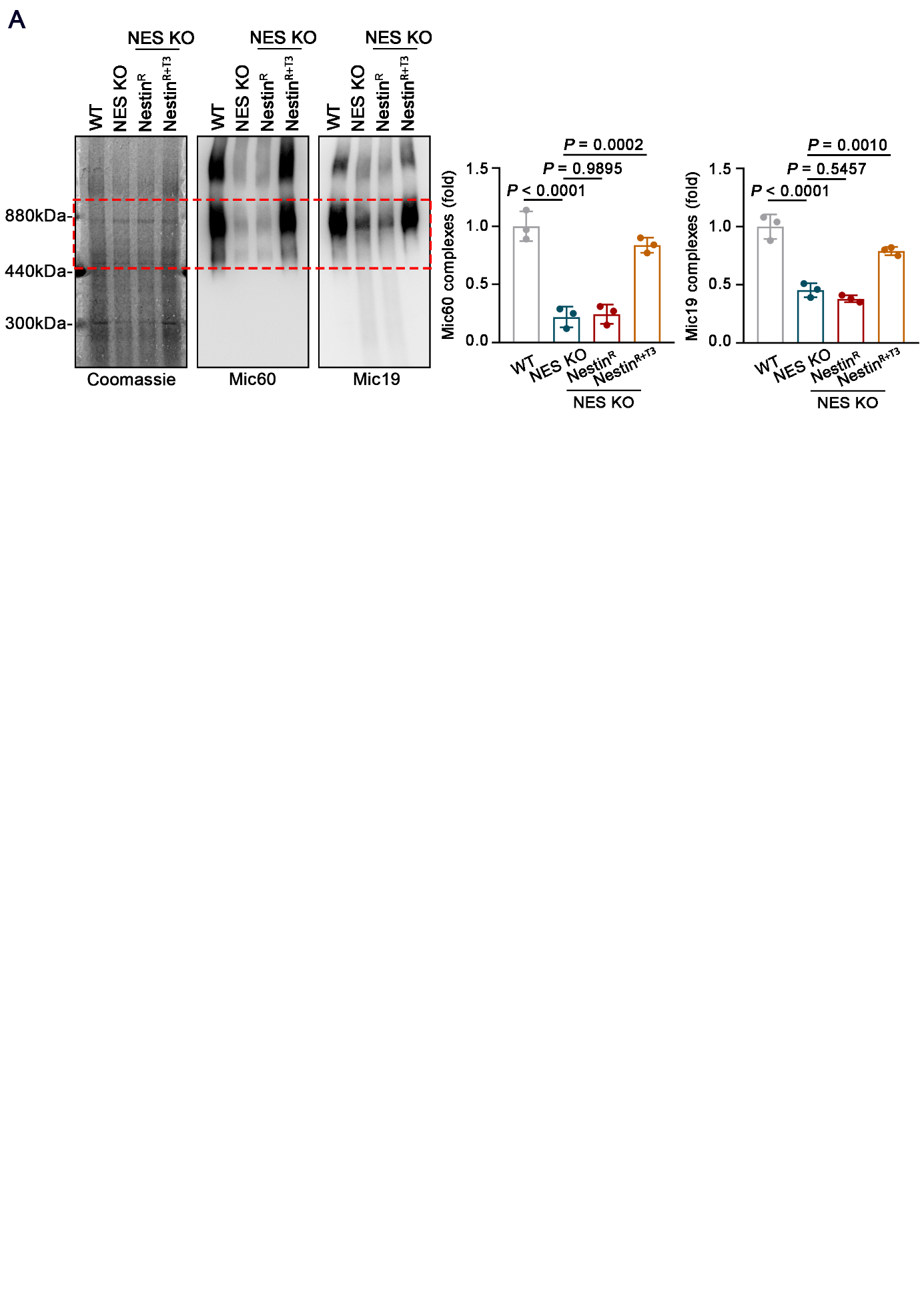


**Figure S6.** Reduced Nestin-Mic60 interaction destabilizes the MICOS complex, related to Figure 5. A) Protein complexes extracted from mitochondria treated as indicated were separated by BN-PAGE, Coomassie stained or transferred onto PVDF membranes, and probed using the indicated antibodies. Data are presented as the means ± SD. Statistical differences determined with one-way ANOVA.


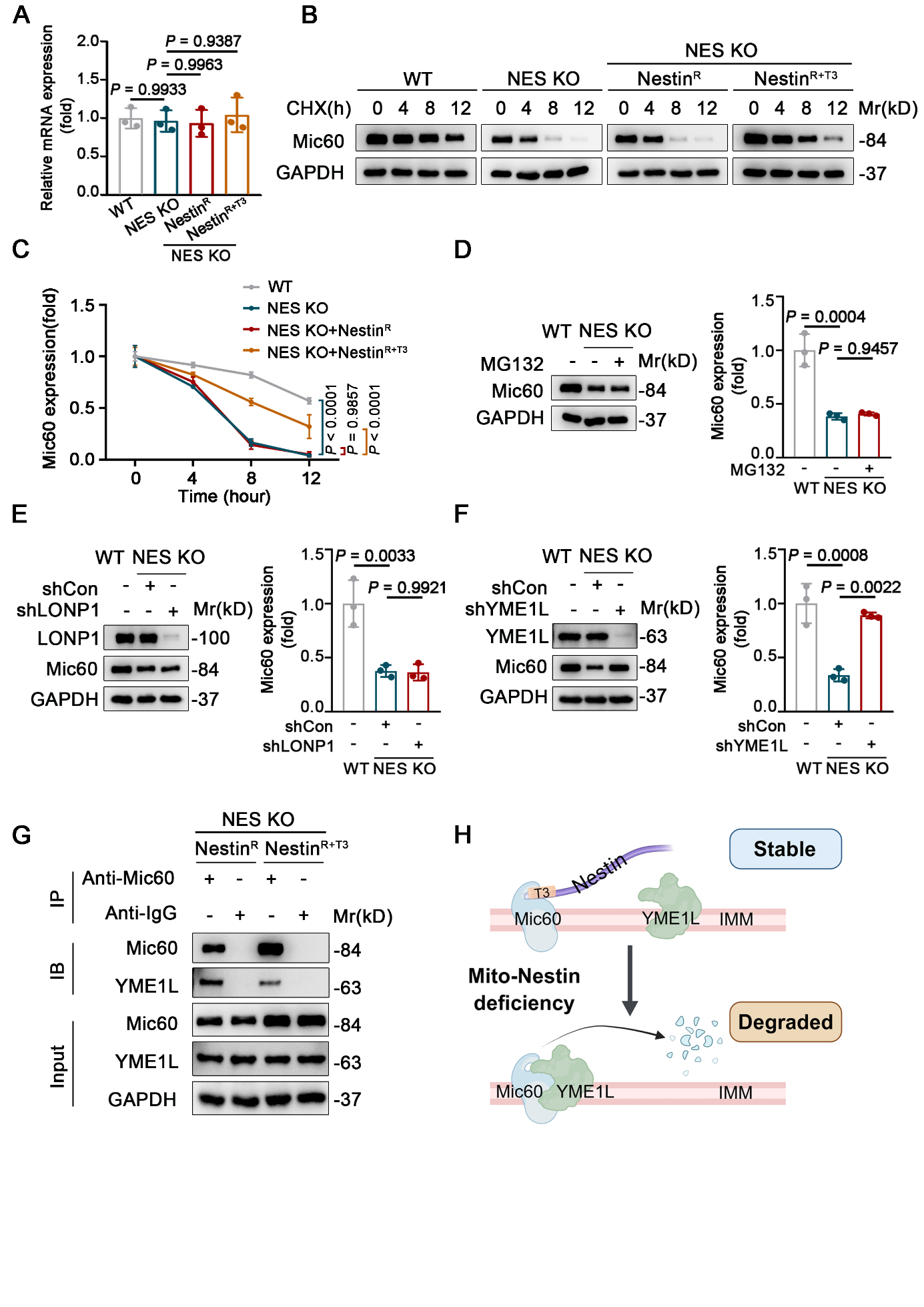


**Figure S7.** Mitochondrial Nestin inhibits YME1L-mediated MIC60 degradation, related to Figure 5. A) mRNA levels of Nestin in indicated hMSCs analyzed by qRT-PCR. B, C) Half-life analysis and quantification of Nestin in indicated hMSCs. All cell groups were treated with cycloheximide (CHX, 50 μg/mL) harvested at the indicated times (0, 4, 8, 12 h after CHX treatment) and subjected to immunoblotting. D) Western blot analysis of Mic60 expression in indicated hMSCs. NES KO hMSCs were untreated or treated with 10 μM of MG132 for 4 h. E) Western blot analysis of Mic60 expression in indicated hMSCs. NES KO hMSCs were transfected with LONP1-knockdown or control plasmids. F) Western blot analysis of Mic60 expression in indicated hMSCs. NES KO hMSCs were transfected with YME1L-knockdown or control plasmids. G) The interactions of Mic60 with YME1L in indicated hMSCs were assessed by co-immunoprecipitation. H) The model of mito-Nestin in regulating Mic60 homeostasis. Under normal condition, mito-Nestin stabilizes Mic60 through direct binding. Upon mito-Nestin deficiency, Mic60 is degraded by mitochondrial protease YME1L. Data are presented as the means ± SD. Statistical differences determined with one-way ANOVA in (A, D, E and F) and two-way ANOVA in (C).


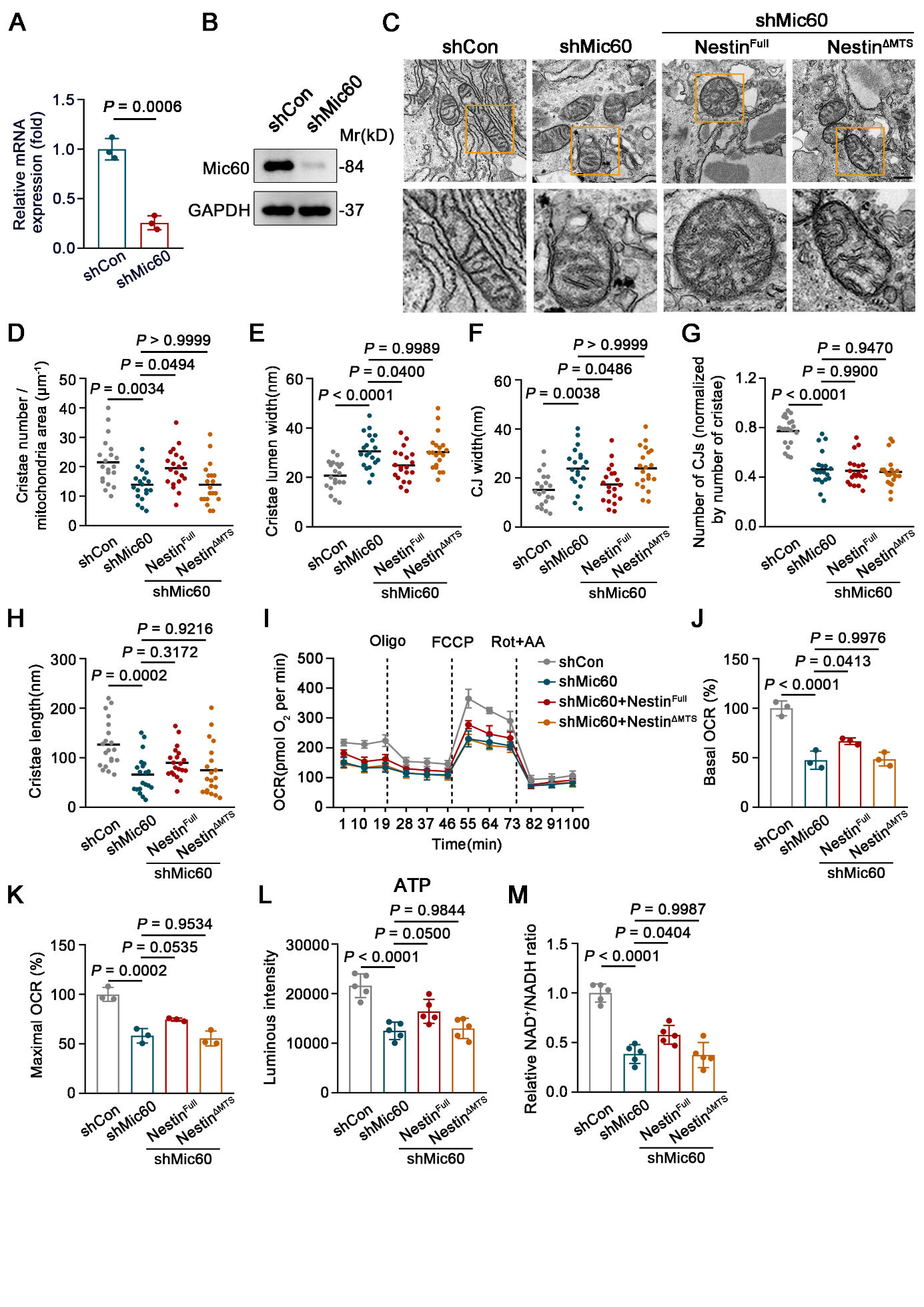


**Figure S8.** Mito-Nestin partially rescues mitochondrial dysfunction and cristae disruption induced by Mic60 deficiency, related to Figure 5. A) mRNA levels of Mic60 in indicated hMSCs analyzed by qRT-PCR. B) Western blot analysis of the expression of Mic60 in indicated hMSCs. C) Representative electron micrographs of mitochondria cristae profiles in indicated hMSCs. Scale bar, 500 nm. D-H) Quantitative analysis of mitochondrial ultrastructure: number of cristae per mitochondria area, cristae lumen width, CJs width, number of CJs and cristae length per crista, as shown in (C). I-K) Seahorse XFe96 analyses of the mitochondrial respiratory capacity of indicated hMSCs (N=3 independent experiments). L, M) ATP production and NAD^+^/NADH ratio detected in indicated hMSCs. Data are presented as the means ± SD. Statistical differences determined with unpaired Student’s t test in (A) and one-way ANOVA in (D-H and J-M).


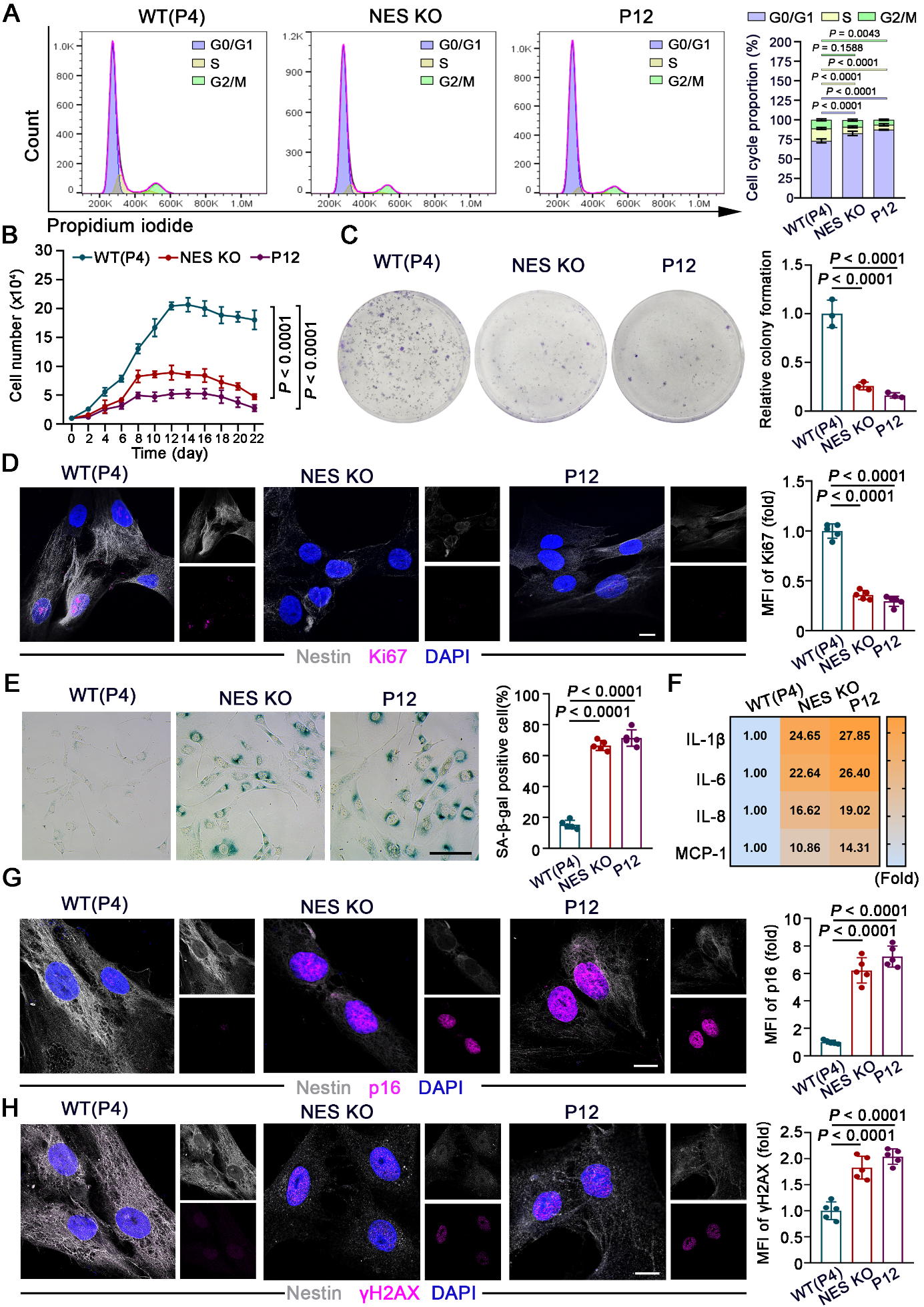


**Figure S9.** Nestin-deficient hMSCs exhibit compromised proliferative ability and accelerated cellular senescence, related to Figure 6. A) PI staining analysis of the cell-cycle percentages of the indicated hMSCs. Cellular senescence progress was evaluated by the percentage of cells in the G0/G1, S, and G2/M phases. B) The proliferation rates of the indicated hMSCs assessed by cell count. C) Representative images of colony formation stained with crystal violet in indicated hMSCs. D) Representative immunostaining images showing the expression of Ki67 in indicated hMSCs. Scale bars, 10μm. E) SA-β-gal staining of indicated hMSCs. Scale bars, 50μm. F) ELISA showing IL-1β, IL-6, IL-8, and MCP1 secretion in indicated hMSCs. Data were shown by fold change and normalized to the WT hMSCs group. G) Representative immunostaining images showing the expression of p16 in indicated hMSCs. Scale bars, 10μm. H) Representative immunostaining images showing the expression of γH2AX in indicated hMSCs. Scale bars, 10μm. Data are presented as the means ± SD. Statistical differences determined with two-way ANOVA in (A and B) and one-way ANOVA in (C, D, E, G and H).


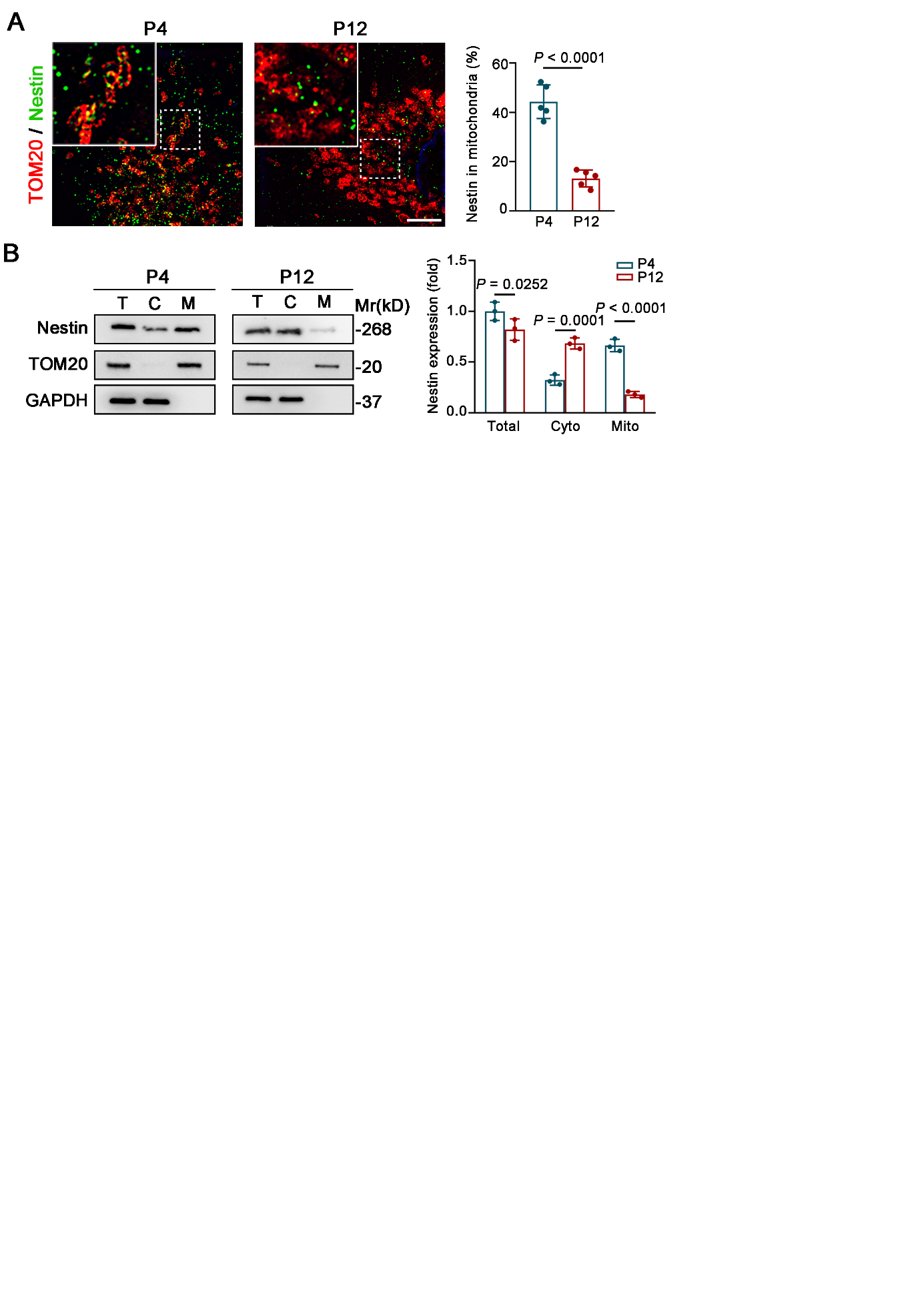


**Figure S10.** Mitochondrial Nestin is reduced in senescent hMSCs, related to Figure 6. A) Representative images of immunofluorescence staining for Nestin with mitochondria (anti-TOM20) in young (P4) and senescent (P12) hMSCs. Scale bar, 5μm. B) Western blot analysis of the expression of Nestin in subcellular fractions including total cellular (T), cytosol (C) and mitochondria (M) in young and senescent hMSCs. Fractionation fidelity was verified by detection of GAPDH in the cytosolic fraction and TOM20 in the mitochondrial fraction. Data are presented as the means ± SD. Statistical differences determined with unpaired Student’s t test in (A) and two-way ANOVA in (B).


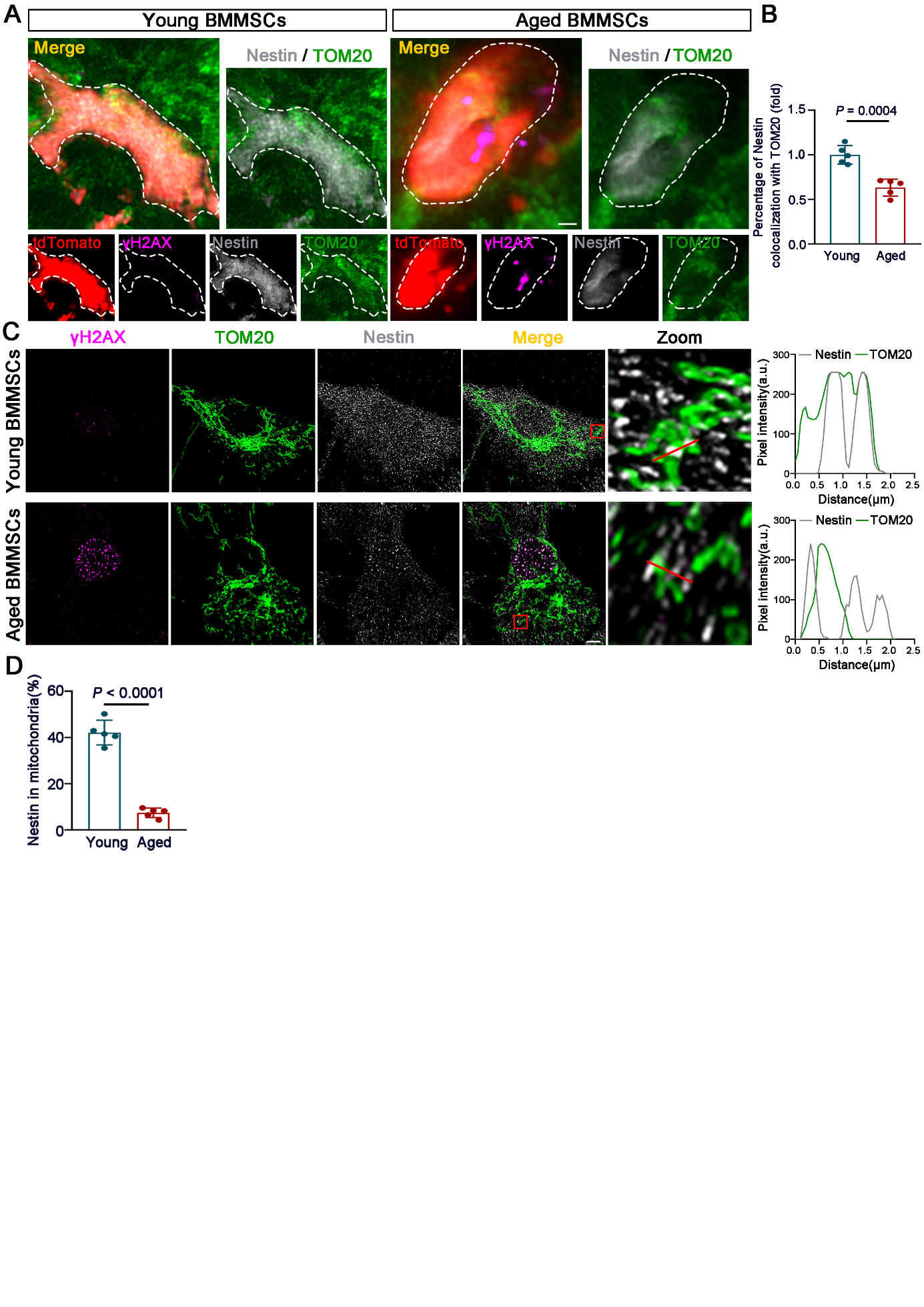


**Figure S11.** In vivo and ex vivo reduction of mito-Nestin in aged BMMSCs, related to Figure 6. A) Representative images of immunofluorescence staining for Nestin, TOM20, γH2AX and tdTomato in bone sections from young (2-month-old) and aged (24-month-old) PDGFRα-creER;Rosa26-CAG-tdTomato mice. Scale bar, 1μm. B) Colocalization analysis of Nestin and TOM20 in indicated BMMSCs. C) Representative immunofluorescence images showing Nestin, TOM20, and γH2AX staining in primary bone marrow mesenchymal stem cells (BMMSCs) isolated from young and aged PDGFRα-creER;Rosa26-CAG-tdTomato mice. Scale bar, 5μm. D) Quantification of the percentage of mitochondria-localized Nestin in indicated BMMSCs. Data are presented as the means ± SD. Statistical differences determined with unpaired Student’s t test.


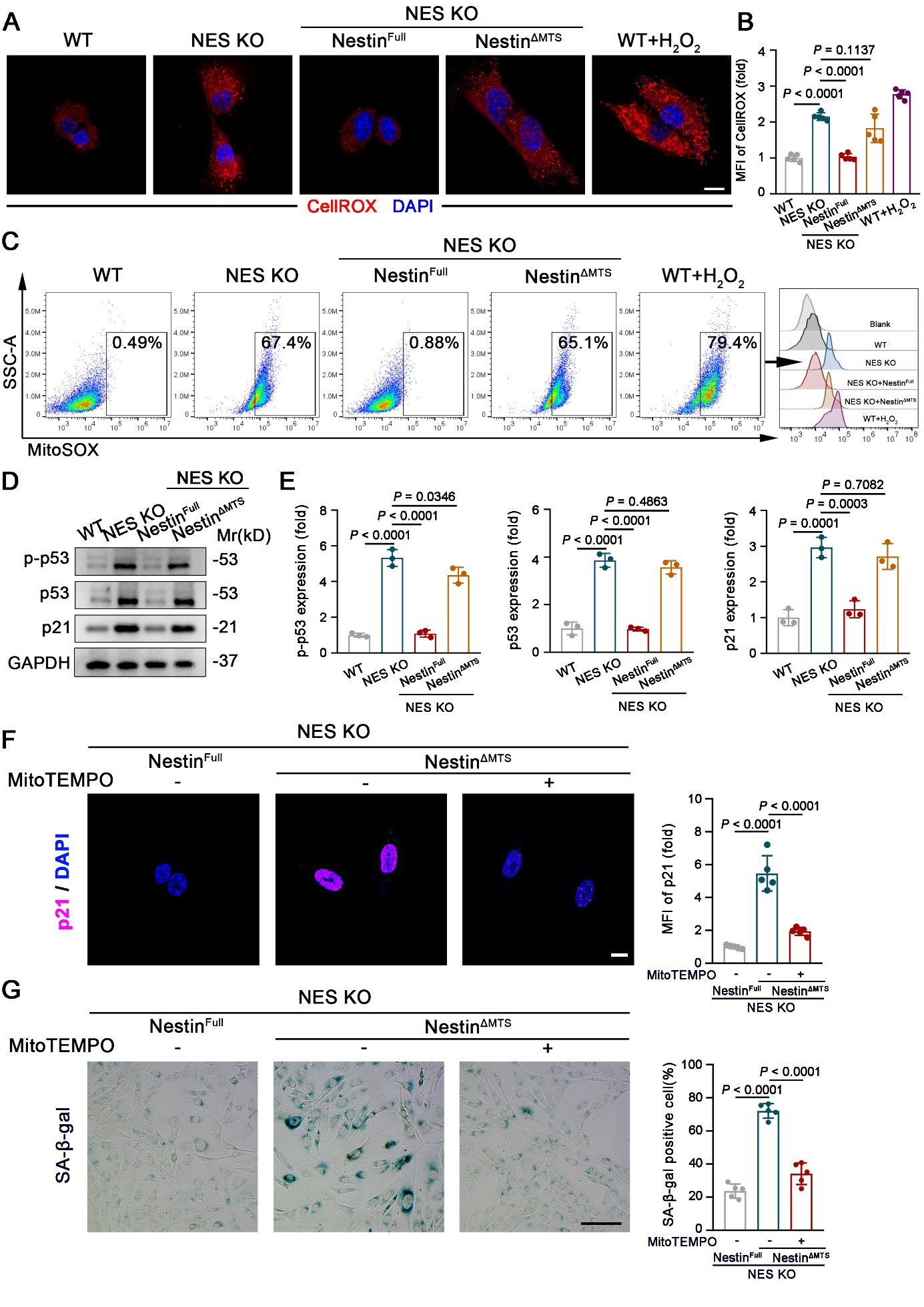


**Figure S12.** Mitochondrial Nestin deficiency elevates ROS and induces p53-p21-mediated senescence, related to Figure 6. A, B) Representative immunostaining images and quantification of intracellular ROS stained with CellRox in indicated hMSCs. 200 μM H_2_O_2_ was the positive control added to the WT group. C) Flow cytometry of mitochodnrial ROS level stained with MitoSOX of the indicated hMSCs. 200 μM H_2_O_2_ was the positive control added to the WT group. D, E) Western blot analysis of p-p53, p53 and p21 in indicated hMSCs. F) Representative immunostaining images showing the expression of p21 in indicated hMSCs. Scale bars, 10μm. G) SA-β-gal staining of indicated hMSCs. Scale bars, 50μm. Data are presented as the means ± SD. Statistical differences determined with one-way ANOVA.

**
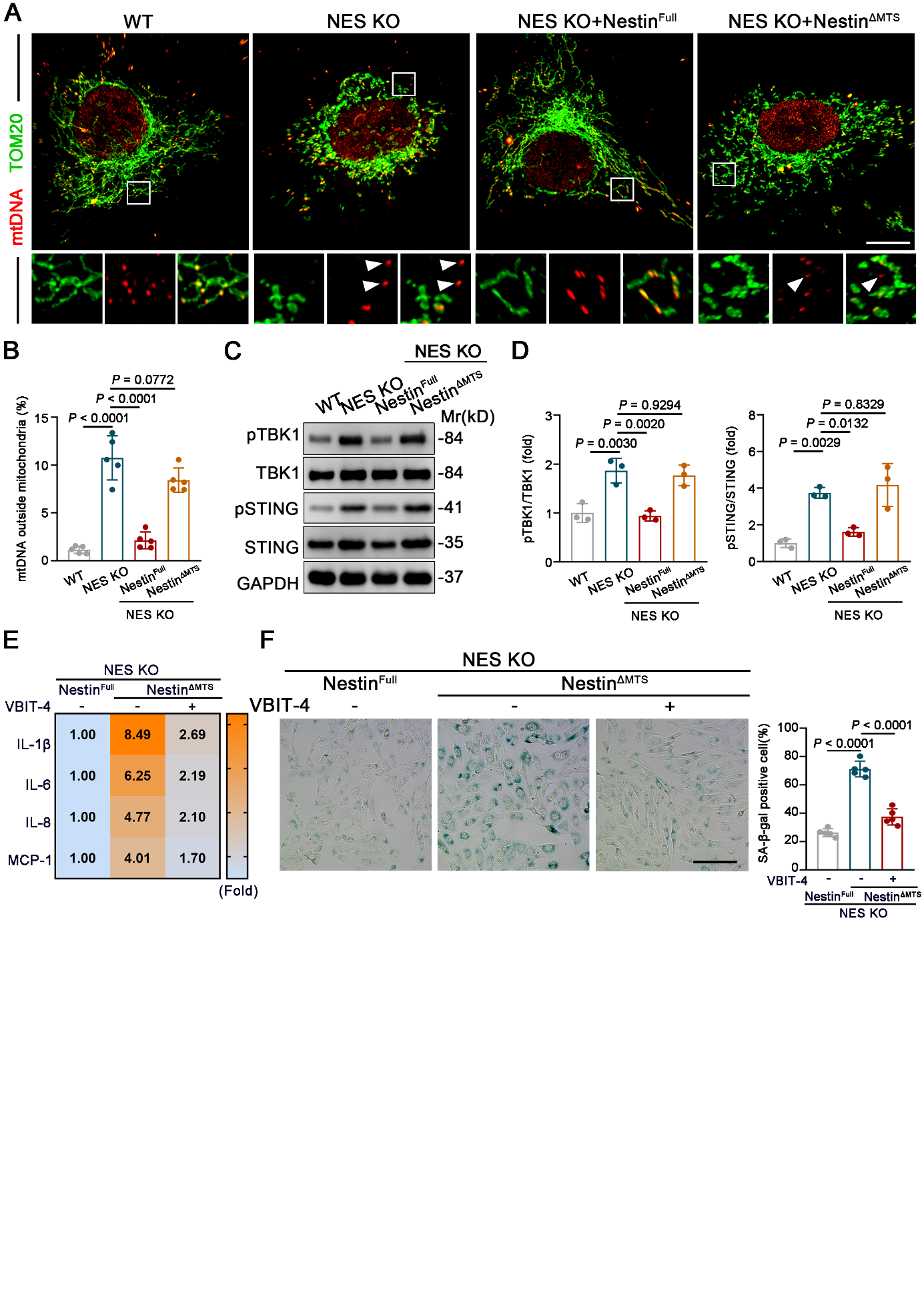
**

**Figure S13.** Mitochondrial Nestin deficiency drives mtDNA leakage and cGAS-STING-mediated senescence, related to Figure 6. A, B) Representative immunostaining images and quantification of mtDNA (red) and TOM20 (green) in indicated hMSCs. Scale bar, 10μm. C, D) Western blot analysis of pTBK1, TBK1, pSTING and STING in indicated hMSCs. E) ELISA showing IL-1β, IL-6, IL-8, and MCP1 secretion in indicated hMSCs. Data were shown by fold change and normalized to the NES KO+Nestin^Full^ hMSCs group. F) SA-β-gal staining of indicated hMSCs. Scale bars, 50μm. Data are presented as the means ± SD. Statistical differences determined with one-way ANOVA.


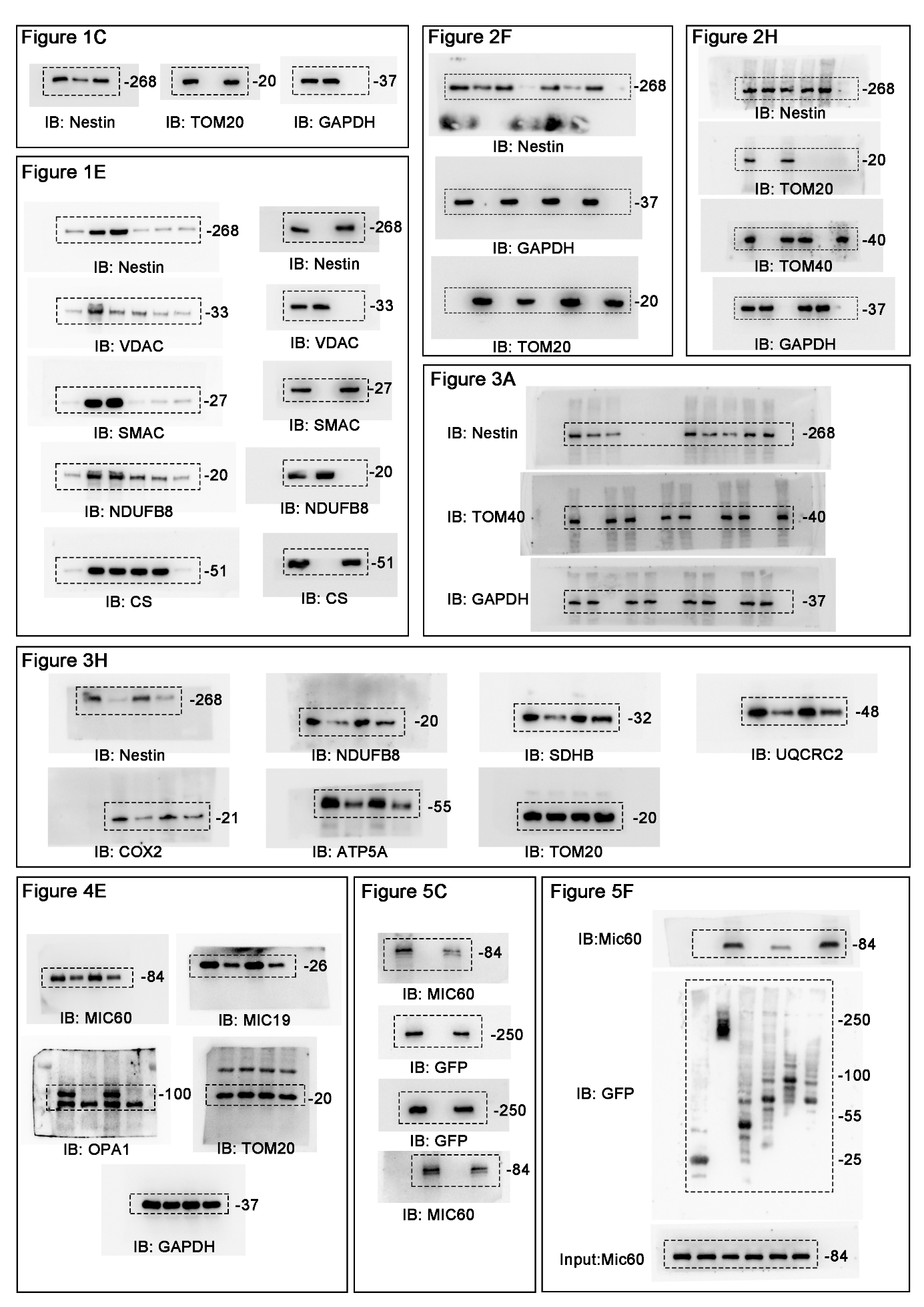


**Figure S14.** Full-length immunoblots, related to Figures 1-5.


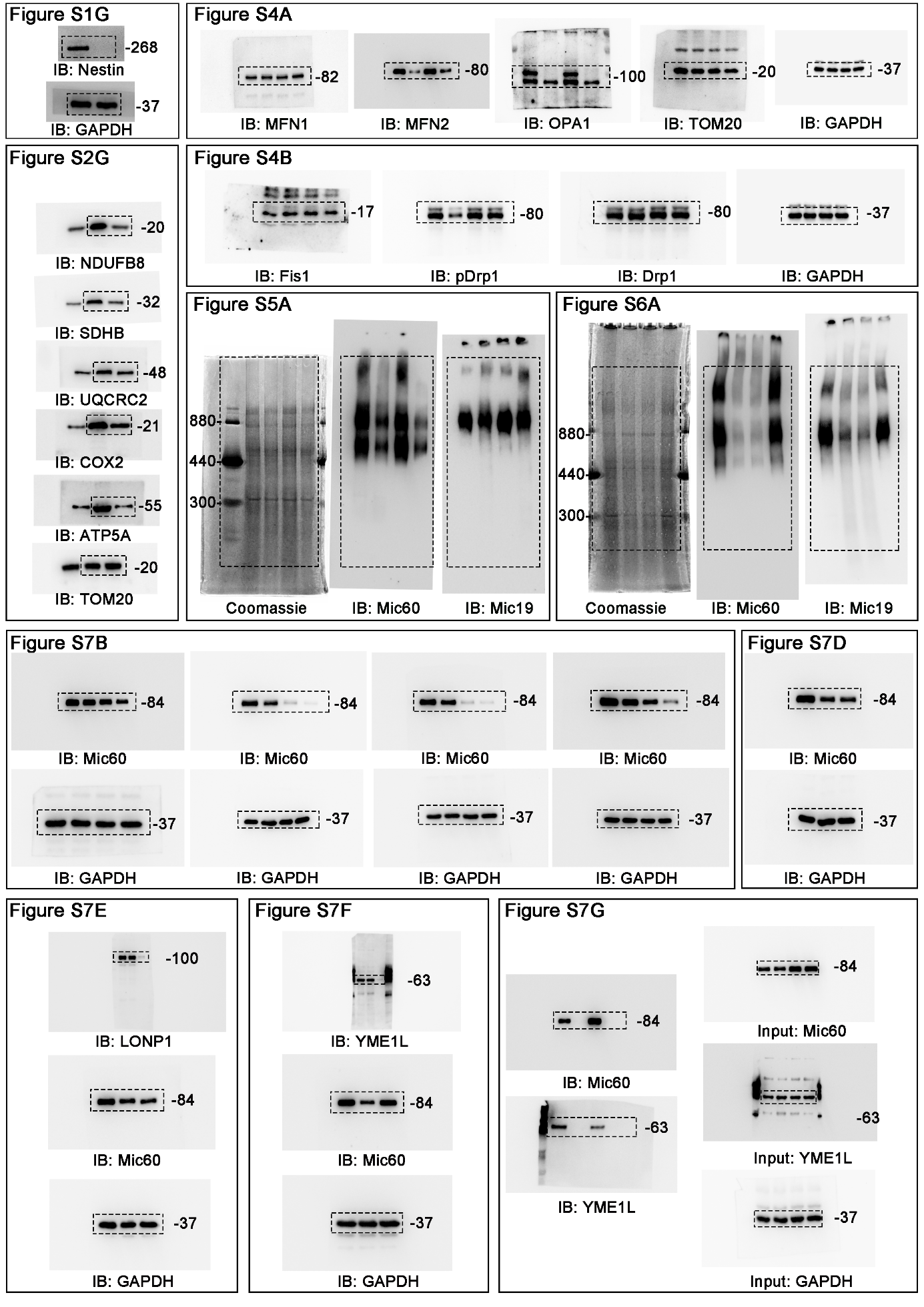


**Figure S15.** Full-length immunoblots, related to Figure S1, Figure S2, and Figures S4-S7.


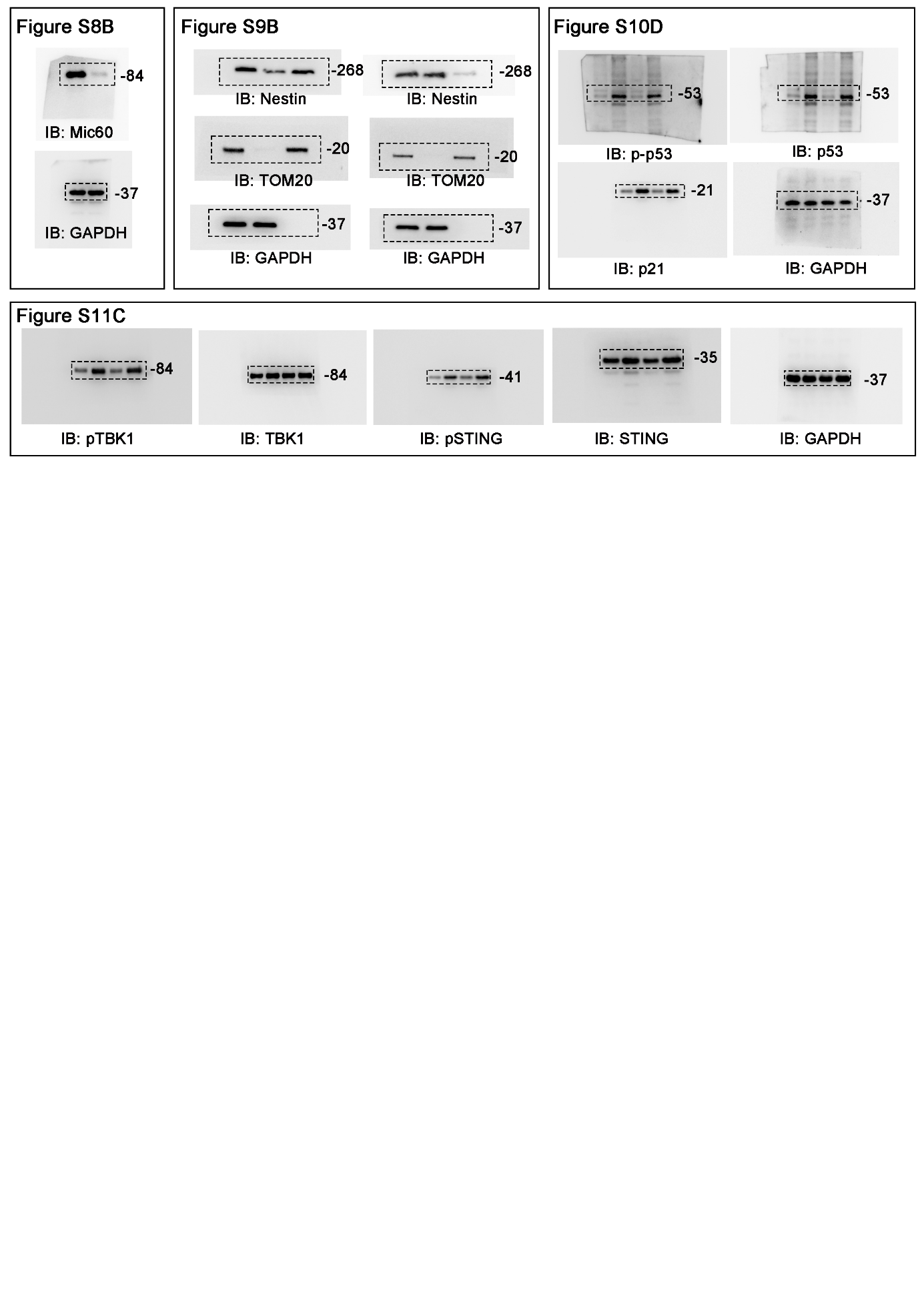


**Figure 16.** Full-length immunoblots, related to Figure S8-S11.

Supplementary Table 1. Primary and secondary antibodies

| **ANTIBODIES** | **SOURCE** | **IDENTIFIER** |
| --- | --- | --- |
| Anti-Nestin | BD Biosciences | Cat#: 611658 |
| Anti-TOM20 | Santa cruz biotechnology | Cat#: sc-11415 |
| Anti-TOM40 | Santa cruz biotechnology | Cat#: sc-365467 |
| Anti-TOM40 | Santa cruz biotechnology | Cat#: sc-11414 |
| Anti-VDAC | Santa cruz biotechnology | Cat#: sc-390996 |
| Anti-SMAC | Santa cruz biotechnology | Cat#: sc-136302 |
| Anti-Citrate synthase | Santa cruz biotechnology | Cat#: sc-390693 |
| Anti-NDUFB8 | Abcam | Cat#: ab192878 |
| Anti-SDHB | Santa cruz biotechnology | Cat#: sc-271548 |
| Anti-UQCRC2 | Santa cruz biotechnology | Cat#: sc-390378 |
| Anti-COX2 | Abcam | Cat#: ab179800 |
| Anti-ATP5A | Santa cruz biotechnology | Cat#: sc-136178 |
| Anti-OPA1 | BD Biosciences | Cat#: 612606 |
| Anti-Mic60 | Abcam | Cat#: ab137057 |
| Anti-Mic19 | Abcam | Cat#: ab224565 |
| Anti-GAPDH | Cell Signaling Technology | Cat#: 2118S |
| Anti-Nestin | Millipore | Cat#: Mab5326 |
| Anti-Nestin | Millipore | Cat#: ABD69 |
| Anti-NDUFB8 | Abcam | Cat#: ab192878 |
| Anti-Nanog | Cell Signaling Technology | Cat#: 4903 |
| Anti-SOX2 | Abcam | Cat#: ab97959 |
| Anti-Oct4 | Santa cruz biotechnology | Cat#: sc-9081 |
| Anti-LC3B | Cell Signaling Technology | Cat#: 83506 |
| Anti-p16-ARC | Abcam | Cat#: ab270058 |
| Anti-p21 | Abcam | Cat#: ab109520 |
| Anti-gamma H2A.X (phospho S139) | Abcam | Cat#: ab26350 |
| Anti-Ki67 | Abcam | Cat#: ab15580 |
| Anti-Mic60 | Abcam | Cat#: ab229653 |
| Anti-GFP | Invitrogen | Cat#: MA5-15256 |
| Anti-Fis1 | Santa cruz biotechnology | Cat#: sc-376447 |
| Anti-MFN1 | Cell Signaling Technology | Cat#: 14739S |
| Anti-MFN2 | Cell Signaling Technology | Cat#: 11925S |
| Anti-Drp1 | Cell Signaling Technology | Cat#: 8570S |
| Anti-pDrp1 | Cell Signaling Technology | Cat#: 4494S |
| Anti-YME1L | Proteintech | Cat#: 11510-1-AP |
| Anti-LONP1 | Proteintech | Cat#: 15440-1-AP |
| Anti- p53 | Santa cruz biotechnology | Cat#: sc-126 |
| **ANTIBODIES** | **SOURCE** | **IDENTIFIER** |
| Anti-TBK1 | Cell Signaling Technology | Cat#: 3504T |
| Anti-STING | Cell Signaling Technology | Cat#: 50494T |
| Anti-Phospho-STING (Ser365) | Cell Signaling Technology | Cat#: 72971T |
| Anti-Phospho-TBK1/NAK (Ser172) | Cell Signaling Technology | Cat#: 5483T |
| Anti-p-p53 | Cell Signaling Technology | Cat#: 9287L |
| Anti-PHB2 | Proteintech | Cat#: 12295-1-AP |
| Anti-DNA | Progen | Cat#: 690014S |
| APC anti-human CD105 | BD Biosciences | Cat#: 562408 |
| APC anti-human CD44 | BD Biosciences | Cat#: 559942 |
| PE anti-human CD73 | BD Biosciences | Cat#: 550257 |
| APC anti-human CD90 | BD Biosciences | Cat#: 559869 |
| BV421 anti-human CD19 | BD Biosciences | Cat#: 562440 |
| PE anti-human CD45 | BioLegend | Cat#: 368510 |
| PECy7 anti-human CD34 | BD Biosciences | Cat#: 560710 |
| Anti-mouse IgG, HRP-linked Antibody | Cell Signaling Technology | Cat#: 7076 |
| Anti-rabbit IgG, HRP-linked Antibody | Cell Signaling Technology | Cat#: 7074 |
| Anti-goat IgG HRP-linked Antibody | Abcam | Cat#: ab6885 |
| Anti-mouse IgG conjugated to Alexa Fluor 488 | Invitrogen | Cat#: A-11001 |
| Anti-mouse IgG conjugated to Alexa Fluor 555 | Invitrogen | Cat#: A-21422 |
| Anti-rabbit IgG conjugated to Alexa Fluor 488 | Invitrogen | Cat#: A-11008 |
| Anti-rabbit IgG conjugated to Alexa Fluor 555 | Invitrogen | Cat#: A-21428 |
| Anti-mouse IgG conjugated to Alexa Fluor 647 | Invitrogen | Cat#: A-21240 |
| Anti-goat IgG conjugated to Alexa Fluor 594 | Invitrogen | Cat#: A-11058 |

**Supplementary Table 2. Primers for qPCR**

| **OLIGONUCLEOTIDIES** | **SOURCE** | **IDENTIFIER** |
| --- | --- | --- |
| Forward: 5′-GAAGGTGAAGGTCGGAGTC-3′  Reverse: 5′-GAAGATGGTGATGGGATTTC-3′ | Sangon Biotech | GAPDH |
| Forward: 5′-CCGCCAAGAAGTATAATATGCGT-3′  Reverse: 5′-TATCCACACGGTTCCTGTTGT-3′ | Sangon Biotech | NDUFB8 |
| Forward: 5′-GACACCAACCTCAATAAG-3′  Reverse: 5′-GATTCATCCTTCTTCTTCAA-3′ | Sangon Biotech | SDHB |
| Forward: 5′-TTCAGCAATTTAGGAACCACCC-3′  Reverse: 5′-GGTCACACTTAATTTGCCACCAA-3′ | Sangon Biotech | UQCRC2 |
| Forward: 5′-ACAGATGCAATTCCCGGACGTCTA-3′  Reverse: 5′-GGCATGAAACTGTGGTTTGCTCCA-3′ | Sangon Biotech | COX2 |
| Forward: 5′-AGGTCCAGGGGTATTGCAG-3′  Reverse: 5′-TCCTCAGGGATCAGTCCATAAC-3′ | Sangon Biotech | ATP5F1 |
| Forward: 5′-CACCCAAGAACAGGGTTTGT-3′  Reverse: 5′-TGGCCATGGGTATGTTGTTA-3′ | Sangon Biotech | mtDNA |
| Forward: 5′-TGCTGTCTCCATGTTTGATGTATCT-3′  Reverse: 5′-TCTCTGCTCCCCACCTCTAAGT-3′ | Sangon Biotech | nDNA |
| Forward: 5′-GGTACTGCATCTACTTCGACCG-3′  Reverse: 5′-TGGTCTACGCCCTTCTCATATTC-3′ | Sangon Biotech | TOM20 |
| Forward: 5′-CTGCTACCCTTGAGACACCTG-3′  Reverse: 5′-GGGCTCTGATCTCTGCATCTAC-3′ | Sangon Biotech | NESTIN |

**Supplementary Table 3. Target sequences of sgRNA and shRNAs**

| **OLIGONUCLEOTIDIES** | **SOURCE** | **IDENTIFIER** |
| --- | --- | --- |
| sgRNA | | |
| 5′-GCTGAGGGGTGGTGCCAAGG-3′ | Constructed in our lab | Nestin sgRNA |
| shRNA | | |
| 5′-GCAGTCTGTTCACAGGTAATT-3′ | Constructed in our lab | ShTOM20 |
| 5′-GCTAGTCCCTGCCTGAATA-3′ | Constructed in our lab | ShNestin |

**Supplementary Table 4. Plasmids**

| **PLASMIDS** | **SOURCE** |
| --- | --- |
| pcDNA3.1-Myc-vector | Invitrogen |
| pcDNA3.1-Myc-Nestin FL (1-1621)-GFP | Constructed in our lab |
| pcDNA3.1-Myc-Nestin R (1-313)-GFP | Constructed in our lab |
| pcDNA3.1-Myc-Nestin R+T1(314-640)-GFP | Constructed in our lab |
| pcDNA3.1-Myc-Nestin R+T2(641-1621)-GFP | Constructed in our lab |
| pcDNA3.1-Myc-Nestin R+T3(1295-1621)-GFP | Constructed in our lab |
| pcDNA3.1-Myc-△MTS Nestin-GFP | Vigene Biosciences |
| pcDNA3.1-Myc-△TRM Nestin-GFP | Vigene Biosciences |
| pcDNA3.1-Myc-△M+T Nestin-GFP | Vigene Biosciences |
| lentiCRISPRv2 vector | Invitrogen |
| pLVTHM vector | Invitrogen |
| pLV/help-SL3 | Invitrogen |
| pLV/help-SL4 | Invitrogen |
| pLV/help-SL5 | Invitrogen |
| pTRE3G-IRES | Clontech |
| pLVXTet3G | Clontech |
| pLVXTet3G- EF1α | Constructed in our lab |
| pTRE3G-IRES- shNestin | Constructed in our lab |
